# Thiolated Bis-*meta*-Carborane: A Molecular Rotor with Conformation-Dependent Dipole Moment

**DOI:** 10.1021/jacsau.5c01656

**Published:** 2026-01-27

**Authors:** Deepak Kumar Patel, Martha Frey, Monika Kučeráková, Jan Macháček, Dmytro Bavol, Julian Picker, Christof Neumann, Zdeněk Bastl, Michal Dušek, Sundargopal Ghosh, Andrey Turchanin, Thalappil Pradeep, Tomas Base

**Affiliations:** † Institute of Inorganic Chemistry, The Czech Academy of Science, 25068 Rez, Czech Republic; ‡ DST Unit of Nanoscience (DST UNS) and Thematic Unit of Excellence (TUE), Department of Chemistry, 37268 Indian Institute of Technology, Madras, Chennai 600036, India; § Institute of Physical Chemistry, 9378 Friedrich Schiller University Jena, 07743 Jena, Germany; ∥ Institute of Physics, The Czech Academy of Science, 182 21 Prague 8, Czech Republic; ⊥ J. Heyrovský Institute of Physical Chemistry, The Czech Academy of Sciences, Dolejškova 2155/3, 18200 Prague 8, Czech Republic

**Keywords:** bis-meta-carborane, dipole, C_2_B_10_H_12_, boron hydride, cluster, molecular rotor, supramolecular structure

## Abstract

The robust structure and tunable properties of 12-vertex carborane clusters make them highly attractive constituents of 2D and 3D self-assembled materials as well as for various potential applications. These molecules offer new possibilities related to their characteristic features such as low conformational freedom, high thermal and chemical stability, and relatively high inherent dipole moment. We synthesized and fully characterized bis-*meta*-carborane-thiol (***mm*** **-SH**), a rod-like molecule with two *meta*-carborane units connected by a single bond, acting as a molecular rotor. In its supramolecular crystal structure, this molecule exhibits an intriguing packing arrangement driven by the interplay between the SH group of a particular conformation, which affects intermolecular hydrogen interaction, and intramolecular dipole–dipole interactions. This interesting interplay manifests itself clearly in the single-crystal supramolecular arrangement, which we have analyzed experimentally as well as computationally. The respective self-assembled monolayer (SAM) of this dipole-responsive carborane constituent was prepared on flat silver and gold substrates and investigated using surface sensitive techniques such as X-ray photoelectron spectroscopy, scanning tunneling microscopy (STM), low-energy electron diffraction (LEED), and ellipsometry. The ***mm-SH*** molecules form a highly ordered structure on the Ag(111) surface, which was measured via LEED and STM with submolecular resolution. It is the first cluster constituent of SAMs that can, by rotation, change the orientation and magnitude of its inherent dipole moment ranging from 0.64 to 3.78 D. Furthermore, the formation of nanomembranes by low-energy electron irradiation of the SAMs (with an effective thickness of 5 ± 1 Å) is presented.

## Graphical abstract

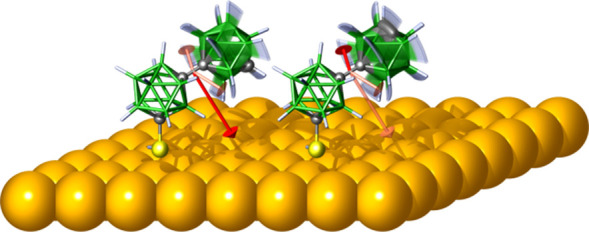

## Introduction

Molecules or molecular fragments that allow free rotation can be considered to be potential molecular rotors, and these systems have attracted significant academic interest. Molecular rotation is a fascinating phenomenon that, much like molecular electronics, highlights the fundamental differences between macroscopic world and the world of molecules.^1–10^ Unlike mechanical objects in the macroscopic world, molecules exhibit various types of molecular interactions from binding (e.g., hydrogen bonds or van der Waals interactions) to nonbinding (e.g., dipole–dipole interactions), all with a potentially significant effect on their rotation.^11–18^ Gaseous molecules exhibit highly dynamic motion, whereas molecular motion in the solid state is much more constrained. Current understanding of molecular rotors largely stems from studies of complex organic structures in solution.^17^ Therefore, it is crucial to investigate molecular behavior both in their free state and when “locked” within various environments, such as two-dimensional (2D) surface arrays or 3D single-crystal supramolecular structures.^15,19,20^ This dual analysis provides deeper insights into the influence of confinement on the rotational dynamics. To achieve collective molecular motion in bulk, amphidynamic materials with rigid stators and rotating components offer anisotropic rotation in the solid state, with dynamics tunable through crystal packing and steric design.^21,22^ Molecular rotation is influenced not only by different types of intermolecular interactions, which can eventually stop their rotational motion, but also by the energy barriers the rotation needs to overcome when passing from one rotamer (conformer) to another.^12,23^ Cage (cluster) molecules have been previously utilized for their high symmetry and robust 3D molecular architectures as parts of molecules in which they function as wheels.^24–28^

Among the robust and highly symmetrical molecules with low conformational freedom are 12-vertex carboranes of the general formula C_2_B_10_H_12_.^29,30^ They exist as three isomers (*ortho-*, *meta-*, and *para-*) with practically identical steric requirements, all derived from icosahedral parental molecule [B_12_H_12_]^2–^ in which two of the BH vertices are replaced for two isolobal CH vertices.^29^ This replacement has only a very small effect on the overall steric requirements of the molecule, and the 12-vertex carborane geometry can still be viewed as an adaption of an almost regular icosahedron, maintaining a nearly identical 3D architecture.^31,32^

The first study of rotation of a single molecule was conducted more than a decade back, but research of rotating molecules in solid state is more challenging.^25^ Unlike in solution, surface-mounted rotors assembled in 2D monolayers, where they are laterally constraint, show great potential in nanoscience due to their functionality at interfaces. Recent use of molecular rotors include measuring microviscosity in liquids and living cells, which brings new possibilities in the areas of chemical flow and biomolecular processes.^27^ Molecular systems, like nanocars and nanowheelbarrows, have been synthesized and studied on surfaces, with *p*-carborane molecule representing the key component.^25^ The first motorized nanocar also used *p*-carborane wheels, which proved more efficient than fullerene-based ones, due to the low rotational barrier of *p*-carborane.^25^ Despite several other examples of carborane-based rotors—such as cobaltabisdicarbollides, phenyl-functionalized carboranes, and fullerene-carborane systems—a thorough understanding of their rotational dynamics and the modulation of nonbonding interactions, such as dipole moments, remains largely unexplored, likely due to experimental challenges. The phenomenon of molecular rotation deserves more attention, as molecular rotors can emulate the function of biological and macroscopic mechanical rotors,^8,10^ converting light,^33–39^ heat,^40,41^ or electrical stimuli^9,42^ into mechanical motion.^5,7,43^ The structural design of cage molecules enables them to function effectively in a wide range of different molecular-scale devices such as propellers,^44^ motors,^45^ Feringa switches,^46^ or sensors.^47^ It was recently revealed that the mobility of nanovehicles is governed by chassis design, surface geometry, and temperature, offering a strategy for selecting optimal designs for targeted applications.^48^

Molecular systems that couple conformational dynamics with tunable dipole moments are very rare, limiting advances in responsive interfaces and dipole-driven devices. In this study, we report on a thiolated bis-*meta*-carborane, 1-(1′-*meta*-carboranyl)-*meta*-carborane-7-thiol, which has two *meta*-carborane clusters linked by a single bond enabling rotation, possesses relatively strong inherent dipole moment, and is equipped with a thiol group to immobilize the molecule on a metal surface into a 2D array. More importantly, this derivative proved very important due to how it assembles into a 3D supramolecular structure, which persists rotational features, and enable to clearly rationalize the interplay between INTERmolecular hydrogen interaction and INTRAmolecular dipole–dipole interaction. Synthesis, all fundamental characterizations as well as experimental and computational analysis of the rotation and supramolecular structure is provided together with essential characterization of this molecule as a constituent of SAMs. This is the first SAM constituent with conformation-dependent dipole moment and we also demonstrate its translation into functional nanomembrane.

## Results and Discussion

Thiolated bis-*meta*-carborane derivative, ***mm*** **-SH** (Figure 1), was synthesized from *meta*-carborane (1,7-C_2_B_10_H_12_) in two steps (Figure S1). In the first step, *meta*-carborane (***m***) was monolithiated and coupled by the addition of CuCl_2_ to form 1,1′-bis-*meta*-carborane (***mm***) similar to that published previously.^49,50^ In the second step, 1,1′-bis-*meta*-carborane was thilolated by lithiation with one equivalent of *n*-BuLi and subsequent addition of sulfur powder followed by quenching with an aqueous solution of hydrochloric acid. The product, denoted here as ***mm*-SH**, was characterized by all standard methods such as multinuclear magnetic resonance (NMR), mass spectrometry, and infrared spectroscopy and also by single-crystal X-ray diffraction analysis. All experimental data including NMR spectra with all resonances assigned to the respective atoms are provided in the Supporting Information (Figures S3–S7 and Table S1). In comparison with all carborane-thiol derivatives that have been reported previously, this bis-*meta*-carborane-thiol is the first example of a carborane SAM constituent that introduces intramolecular dipole–dipole interaction as an inherent part of the molecule, and we report here on how its rotation translates into the single-crystal supramolecular packing, which exhibits a very specific intermolecular hydrogen interaction. In the next text, we first address the molecular geometry including the conformational analysis, then we demonstrate how the molecules assemble into a single-crystal 3-dimensional supramolecular structure, and then we provide the analysis of this derivative as a constituent of self-assembled monolayers (SAMs) characterized using X-ray photoelectron spectroscopy (XPS), scanning tunneling microscope (STM), and low-energy electron diffraction (LEED). Finally, the formation of nanomembranes formed by low-energy electron irradiation is presented.

**Figure 1:**
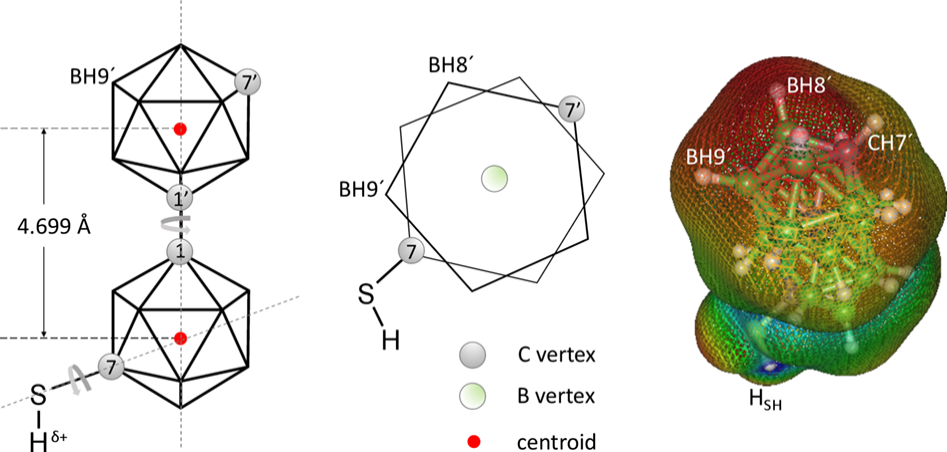
Schematic representation of ***mm*** **-SH** molecule (on the left), schematic top view along the C1–C1′ axis depicting only the top and bottom pentagons with the C7 and C7′ carbon positions and the capping green boron vertex in the center (middle schematic), and molecular electrostatic potential mapped on an isosurface of the electron density (on the right).

### Molecular Geometry

The molecular structure of ***mm*** **-SH** was analyzed computationally, and selected intramolecular distances were compared to similar ones in *meta*-carborane (***m***), *meta*-carborane-1-thiol (***m1***), and 1,1′-bis-*meta*-carborane (***mm***). This comparison shows clearly the effect of a substituent (of both a *meta*-carboranyl moiety as well as of a thiol group) on the selected distances inside the cluster. All of these derivatives have the substituent attached to the carbon atom, Table 1 shows distances between the centroid of the respective *meta*-carborane cluster (Cn or Cn′) and the particular carbon vertex, labeled here for simplicity as C1, C2, C1′, and C2′. Schematic representation of all four molecules with this simplified numbering is shown in the Supporting Information, Figure S2. In agreement with previous reports, the substituent generally increases the distances in the carborane cage.^51^ It is interesting to note that the attachment of an SH group to a carbon vertex increases the distance between the respective *meta*-carborane centroid and the particular carbon vertex from 1.54 Å in ***m*** to 1.57 Å in ***m1***, and from 1.53 Å in ***mm*** to 1.56 Å in ***mm-SH***, i.e., for 0.03 Å in both cases. The attachment of a *meta*-carboranyl moiety leads to the centroid–carbon vertex distance increase of about 0.04 Å (from 1.54 Å observed in ***m*** to 1.58 Å in ***mm***). Although these substituent-caused changes might seem very small, they have been shown to have more apparent effect in lowering the temperature that is necessary for carborane skeletal rearrangement.^51^ There is another aspect that is more important for this study, which relates to the SH group effect on the electron density distribution over the cluster surface. Figure 1 (on the right) shows the molecular electrostatic potential mapped on the isosurface of the electron density as an essential analysis providing rationalization of the specific interactions (and thus also arrangement) these molecules display in the 3D single-crystal supramolecular structure. Red areas represent the lowest electrostatic potential; they are localized mainly on and around the vertices BH8′ and BH9′, and reveal which side of the molecule will have affinity toward areas of the highest electrostatic potential localized on the acidic thiol hydrogen atom, −SH, which has partially positive charge, δ+, (shown in blue) and protrudes out of the cluster.

**Table 1:** Selected Centroid-Vertex Distances (Å) in ***mm-SH***, and, for Comparison, Also in ***mm***, ***m1***, and ***m*** ^a^

	*d*(Cn···C1)	*d*(Cn···C2)	*d*(Cn′···C1′)	*d*(Cn′···C2′)
***m***	1.54	1.54		
***m*1**	1.54	1.57		
***mm***	1.58	1.53	1.58	1.53
***mm*-SH**	1.58	1.56	1.58	1.53

a Cn1 and Cn2 are centroids of the *meta*-carborane clusters as defined in the Supporting Information Figure S2. Centroids are calculated from the 12 respective vertices (10 boron and 2 carbon).

### Conformational Analysis

Figure 1 shows a schematic representation of the molecule of ***mm-SH*** with two depicted rotations, one around the bond between the two *meta*-carborane cages and also rotation of the SH group. We carried out conformational analysis of both ***mm*** and ***mm-SH***. The former one, ***mm***, has three symmetrically unique rotamers of almost identical energies, the centrosymmetric one with the torsion angle C7–C1–C1′–C7′ of 180° being the most stable (Table 2, Figure 2, top). The rotational barriers between the rotamers are very low, less than 30 kJ/mol, and free rotation may be expected at laboratory temperature (300 K).

**Table 2:** Torsion Angles, Energies, and Boltzmann Factors at Low Temperature (95 K) and Laboratory Temperature (300 K) for the Rotamers and Transition Conformations of 1,1′-bis-*meta*-carborane (***mm***)

		e^–Δ*E*/*RT*^	
torsion angle [°]	Δ*E* [kJ/mol]	95 K	300 K
0.000	29.37	3.12 × 10^–17^	7.68 × 10^–06^
36.210	1.76	0.10	0.49
71.509	28.03	1.77 × 10^–16^	1.32 × 10^–05^
108.705	0.60	0.46	0.79
143.621	26.95	7.15 × 10^–16^	2.03 × 10^–05^
180.000	0.00	1	1

**Figure 2:**
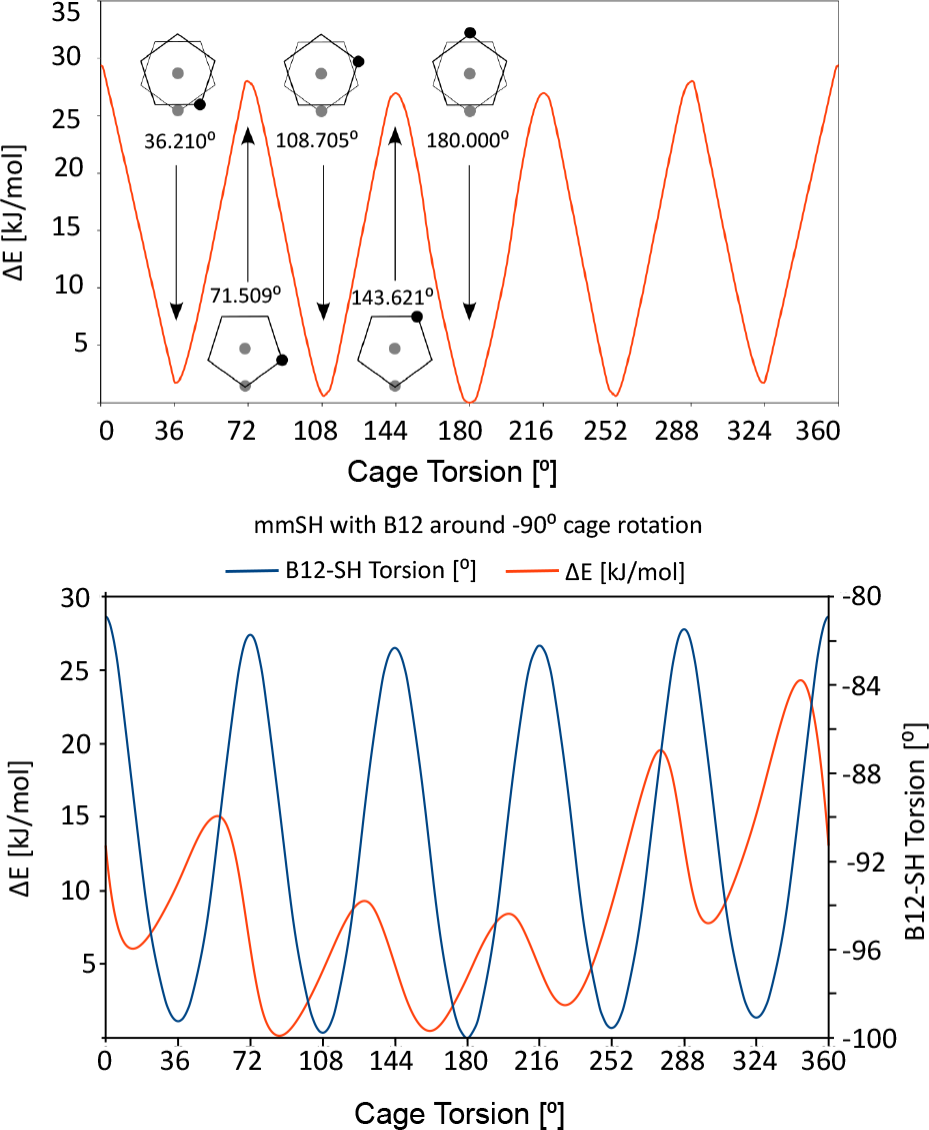
Rotation profile of ***mm*** (top) and ***mm-SH*** (bottom). Top: *mm* has three nearly identical rotamers, with the most stable having a C7–C1–C1′-C7′ torsion angle of 180°. Low rotational barriers (<30 kJ/mol) allow free rotation at room temperature. Bottom: ***mm-SH*** shows similar behavior, with the SH group’s H–S–C7-B12 torsion angle adjusting to the cage conformation (∼15° range) with low rotational barriers (<30 kJ/mol), also allowing free rotation at room temperature.

Conformational analysis of ***mm-SH*** is more complicated due to the second axis of rotation (−S-H group rotation). Figure 2 (bottom) therefore shows the C7–C1–C1′–C7′ torsion angle of *meta*-carborane cages accompanied by the H–S–C7–B12 torsion angle values of the SH group at about 90°, which shows that the SH group torsion angle adjusts to the particular orientation of the two *meta*-carborane cages within the range of approximately 15°. Although it shows a particular SH group conformation as a function of the conformation of the cages, the energy differences of all the SH group conformers are so small that it implies completely free rotation at room temperature (Table 3). Like in the case of ***mm***, the conformer of ***mm-SH*** with the C7–C1–C1′–C7′ torsion angle of 180° is the most stable one. The other conformers are nevertheless very similar in energy, and the rotational barriers between the rotamers of ***mm-SH*** are less than 30 kJ/mol, which also implies practically free rotation at the laboratory temperature (300 K). It is important to note that the 3D aromaticity remain largely localized within each carborane unit and does not extend significantly through the 1–1′ connecting bond.^52^

**Table 3:** Torsion Angles (C7–C1–C1′–C7′ Torsion Angle of *Meta*-Carborane Cages and the H–S–C7–B12 Torsion Angle), Energies, and Boltzmann Factors at Low Temperature (95 K) and Laboratory Temperature (300 K) for the Rotamers and Transition Conformations of ***mm-SH***

torsion angle [°]		Δ*E* [kJ/mol]	e^–Δ*E*/*RT*^	
C7–C1–C1′–C7′	H–S–C7–B12		95 K	300 K
–0.008	–91.284	28.62	8.23 × 10^–17^	1.03 × 10^–05^
0.008	91.284	28.62	8.23 × 10^–17^	1.03 × 10^–05^
36.033	89.892	1.38	0.17	0.58
36.119	–92.963	1.13	0.23	0.64
71.919	91.310	27.76	2.51 × 10^–16^	1.46 × 10^–05^
71.955	–95.875	27.39	4.04 × 10^–16^	1.70 × 10^–05^
108.032	–96.975	0.35	0.64	0.87
108.052	93.998	0.67	0.42	0.76
143.881	96.843	26.65	1.06 × 10^–15^	2.28 × 10^–05^
143.951	–96.667	26.50	1.28 × 10^–15^	2.42 × 10^–05^
179.988	–97.266	0.00	1	1
179.988	97.265	0.00	1	1

### Single-Crystal Supramolecular Arrangement

Single-crystal X-ray diffraction study of ***mm-SH*** revealed an interesting supramolecular structure. Figure 3 shows a colored ORTEP picture of the structure. Positions of all the heavier atoms (S, B, and C) in the molecules show specific disorders in the structure. The supramolecular array consists of molecules linked via interactions involving their thiol (−SH) groups into two-dimensional sheets. A fragment of one of these sheets is depicted in Figure 3. In the supramolecular 3D array, these sheets are rotated 90° and stacked on top of each other, effectively filling up the seemingly free space between the molecules shown in Figure 3. Stacking of the sheets is presented in Figure S10. From a crystallographic perspective, this arrangement presents a particularly intriguing and challenging structure to resolve.

**Figure 3:**
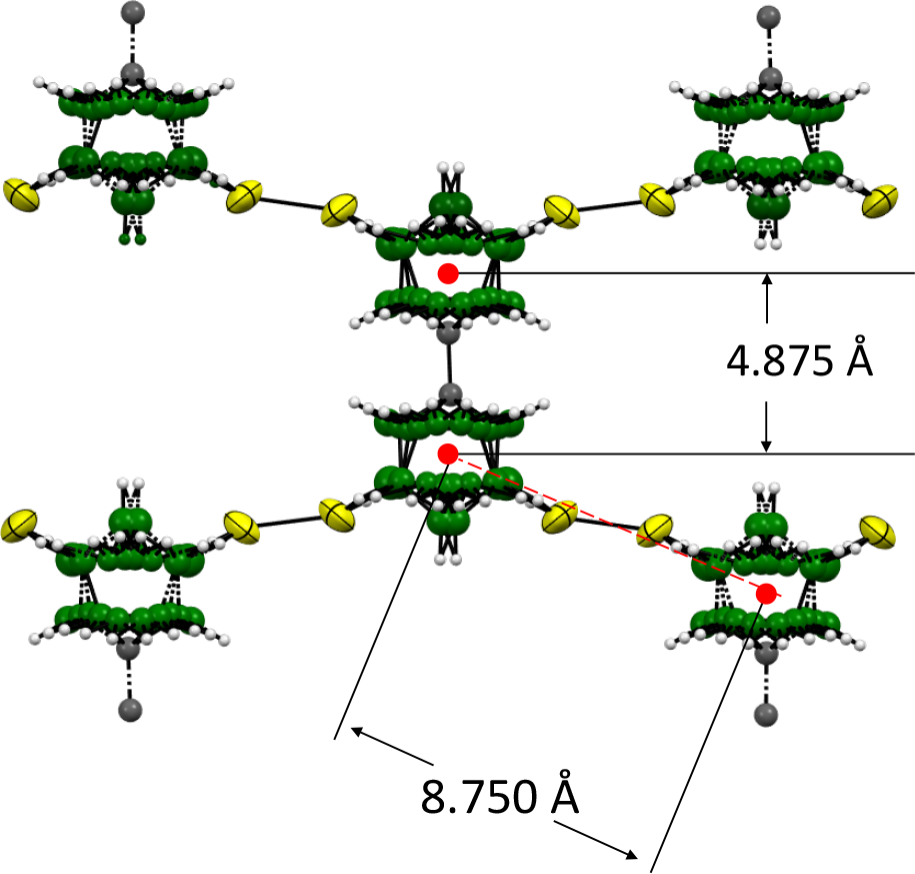
Crystallographically determined structure of 1-(1′-*meta*-carboranyl)-*meta*-carborane-7-thiol (***mm-SH***). Red points show the positions of centroids of the respective cluster parts of the molecules.

The positively charged hydrogen atom in the SH group enables two types of interactions: first, hydrogen bond between two SH groups, i.e., −SH···S­(H)–, and second, hydrogen bond interaction between the SH group and hydridic HB vertices bearing partial negative charge, i.e., −SH···HB–. Both of these interactions have been previously reported, and their examples are shown in the Supporting Information (Figures S11 and S12).

The thiol sulfur atoms (−SH) are crystallographically disordered too, showing four equivalent positions between the carborane cages (Figure 4). Nevertheless, neither of the distances between the positions of sulfur atoms in ***mm-SH*** correspond to the typical distance of sulfur atoms as in an −SH···S­(H)– hydrogen interaction (approximately 4 Å, Figure S11), and this type of interaction can therefore be excluded from further consideration.

**Figure 4:**
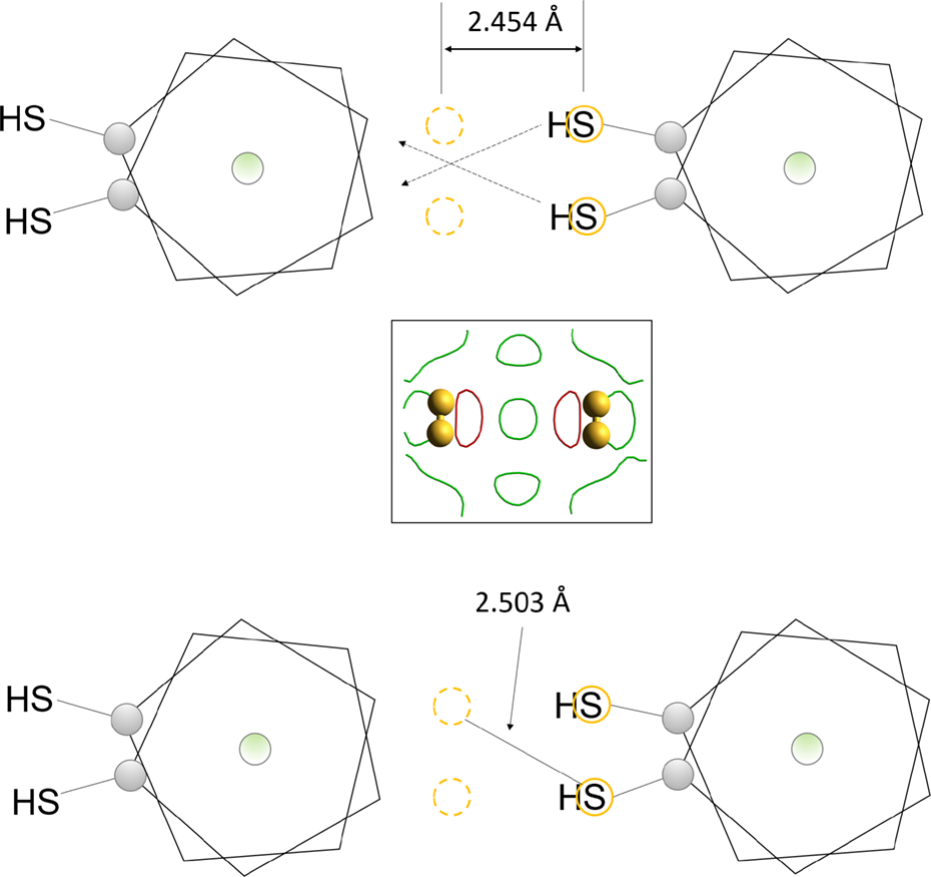
Positions of sulfur atoms (yellow solid and dashed circles) connecting *meta*-carborane cages by hydrogen −SH···HB– interactions. The inset in the middle shows an experimental difference Fourier map of a section defined by the sulfur atoms, merging the electron density 0.25 Å above and below the plane.

On the other hand, there is a very good match between −SH···HB– distances observed in the ***mm-SH*** supramolecular structure and this interaction reported previously (Figure S12). The molecule of ***mm-SH*** shows several BH vertices bearing a partially negative charge on the respective hydrogen atom and thus suitable for the interaction with an SH hydrogen atom of another molecule. Although many of them are located on both the thiol-substituted and the unsubstituted *meta*-carborane cage, those that are slightly further away from the CH vertex and from the SH group are more likely to be involved in this interaction. This leads to the supramolecular structure shown schematically in Figures 4 and 5, with various molecular conformations depicted by different possible positions of carbon atoms.

**Figure 5:**
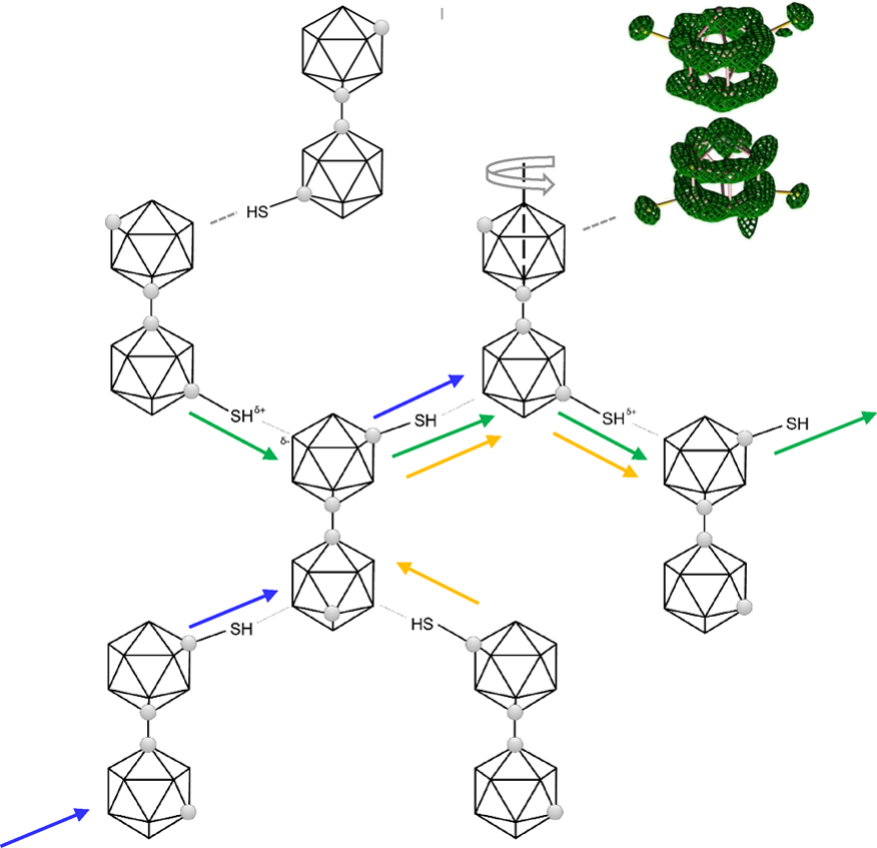
Molecules of ***mm-SH*** associated with hydrogen −SH···HB– interactions in a 2D sheet. Arrows of different colors (green, blue, and orange) depict some of the possible propagations of the −SH···HB– chain of interactions throughout this 2D sheet-like arrangement. Hydrogen atoms in the carborane cluster vertices are omitted for clarity. The top right part of the figure shows the experimental electron density as measured using X-ray diffraction.

It is important to note that although the schematic representations depict particular conformations of the molecules, practically continuous experimental electron density has been measured using X-ray diffraction (Figure 5, top right), which fits practically the free rotation of the molecule. Also, multiple different ways of propagation of the −SH···HB– interaction throughout the 2D sheet of the supramolecular structure are shown in Figure 5. The free rotation is supported by practically identical energies of different rotamers as well as by the low energy barriers of the transition states, as shown in Figure 2.

### Conformation-Dependent Dipole Moment

The conformation-dependent dipole moment of ***mm*-SH** plays a crucial role in determining its orientation, intermolecular interactions, and overall organization within the SAMs. As illustrated in Figure 6, three representative rotamers demonstrate how subtle changes in the molecular conformation significantly influence the dipole moment. The most stable rotamer (with the torsion angle C7–C1–C1′–C7′ of 180°) shows the dipole moment value of only 1.13 D due to almost parallel and opposite orientation of the two *meta*-carborane units in the molecule of ***mm-SH***. Fittingly, rotamer with the torsion angle of 36°, in which both *meta-*carborane units of the molecule are pointing toward each other, exhibits the dipole moment of 3.78 D (the greatest value of all the conformers). It is important to note that the transition state with the torsion angle of 144° displays the value of dipole moment of only 0.64 D and shows also the lowest limit of the range (0.64–3.78 D) in which the dipole can continuously alternate in this molecule due to its rotation. Complete set of dipole moment values for all the conformers and transition states are provided in the Supporting Information Table S4.

**Figure 6:**
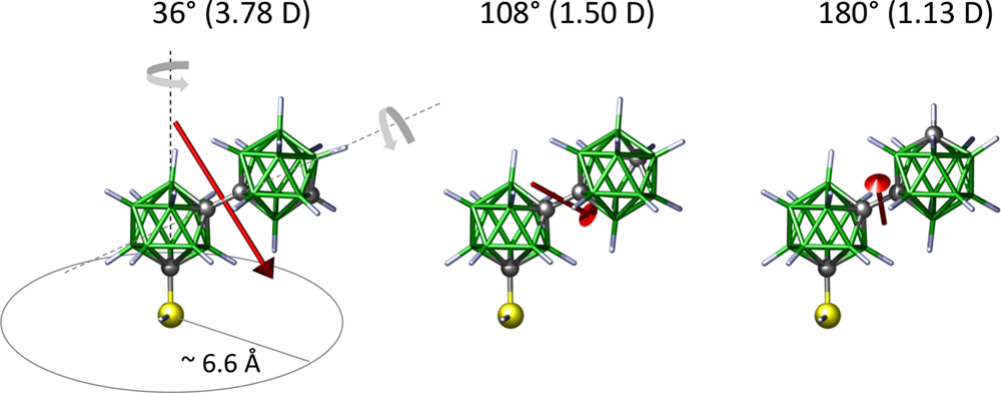
Top: Three conformers (36°, 108°, and 180°) have red arrows showing the projection of their respective dipole moment orientation and magnitude. On the left: schematic of free rotation around two axes of the molecule illustrating their respective lateral steric requirements and flexibility of dipole moment changes.

### X-ray Photoelectron Spectroscopy Analysis of the Clusters

It is worth noting that one of the fundamental challenges in studying crystalline thiols of (car)­borane clusters using XPS is their high volatility. Understanding the binding energy (BE) of free thiols, as well as their behavior when bound to metal surfaces, is crucial. To explore this further, we conducted XPS analysis on 3D ***mm*** **-SH** crystalline solid. While B-SH functionalized **O9,12**-carboranethiols in crystalline form have been previously reported,^51^ this study represents the first successful measurement of a pure crystalline C–SH functionalized carborane using XPS (Figure S14), enabled by its lower volatility compared to parent carborane cages, as shown using DTA analysis (Figure S9). The relatively greater mass of ***mm*** **-SH** and the strong dipole moment of **O9,12** make these two derivatives similarly low in volatility. Notably, this is one of the only two reported instances of free carboranethiols, with ***mm*** **-SH** featuring a C–S–H bond and **O9,12** containing a B–S–H bond. This provided a unique opportunity to precisely examine the electron-accepting and electron-donating properties of carborane cages via comparing the BE values of S 2p electrons in these free thiol groups. As shown in Figure S14, the high-resolution S 2p spectrum reveals one type of sulfur with the BE value of S 2p_3/2_ component at 163.5 eV. The BE differences observed, when measured under identical conditions, for the respective B-SH derivative **O9,12** show the value of 162.9 eV. This significant difference of ∼0.6 eV manifests the differences between boron and carbon vertices of a carborane cage, and its respective electron-donating (**O9,12**) or electron-accepting (***mm*** **-SH**) properties. With this understanding of the binding energies for the free thiols, it becomes important to compare these values when the thiols are bound to a metal surface.

### SAMs on Metal Surfaces: STM, LEED, and XPS Analysis

The molecule of ***mm-SH*** represents a more structurally complex and challenging carborane-based constituent of SAMs compared to previously reported systems.^48^ Conventional carborane SAMs typically exhibit laterally isotropic steric requirements, low conformational freedom, and, consequently, long-range order with a low number and variety of defects. However, in the case of ***mm-SH***, these features are influenced by its unique molecular geometry with high aspect ratio and conformation-dependent dipole moment. While the 2D array effectively suppresses the swinging rotation of the molecule along the S–C bond, it does not show any evidence of disabling the conformation-dependent rotation along the C1–C1′ bond connecting the carborane subunits. To examine how restricted rotational motion impacts the packing and defect formation in these SAMs, SAMs of ***mm-SH*** were prepared using both solution-phase deposition under ambient conditions on Au/mica substrates and physical vapor deposition (PVD) under ultrahigh vacuum (UHV) on Ag(111) substrate. SAMs formed via PVD exhibited well-ordered and periodic arrangements of ***mm-SH*** molecules on the surface. In contrast, SAMs prepared from solution under ambient conditions displayed lower surface coverage and less structural order.

The successful formation of these SAMs was investigated by using XPS. Figure 7 shows the XP spectra of these SAMs before and after irradiation with low-energy electrons. The S 2p spectrum of the SAM exhibits one doublet at BEs of 161.9 eV (S 2p_3/2_) and 163.1 eV (S 2p_1/2_), which can be attributed to the formation of thiolate bonds on the silver surface. Similar BEs have been observed in other carborane- and borane-based SAMs.^19,53^ The B 1s spectrum shows a single peak at 189.5 eV, typical for B–B bonds in carborane clusters. The C 1s spectrum shows a component at a BE of 286.1 eV that can be attributed to the carbon atoms in the carborane cage. Additionally, there is a minor peak present at 284.0 eV that is typical for C–C and C–H bonds originating from hydrocarbon impurities. The high quality of the formed SAMs is further confirmed by the absence of an O 1s signal. The elemental C/S/B stoichiometry ratio is found to be (2.1 ± 0.4):(0.8 ± 0.2):10. Sulfur and carbon appear weaker than the nominal stoichiometry (4:1:10), since the boron cages above the thiol group and the lower carbon atom weaken the signal by shielding them. The effective thickness of the SAM is determined to be 5 ± 2 Å based on the attenuation of the Ag 3d_5/2_ signal, closely matching the estimated height of the molecule on the silver surface. These results confirm the successful SAM formation.

**Figure 7:**
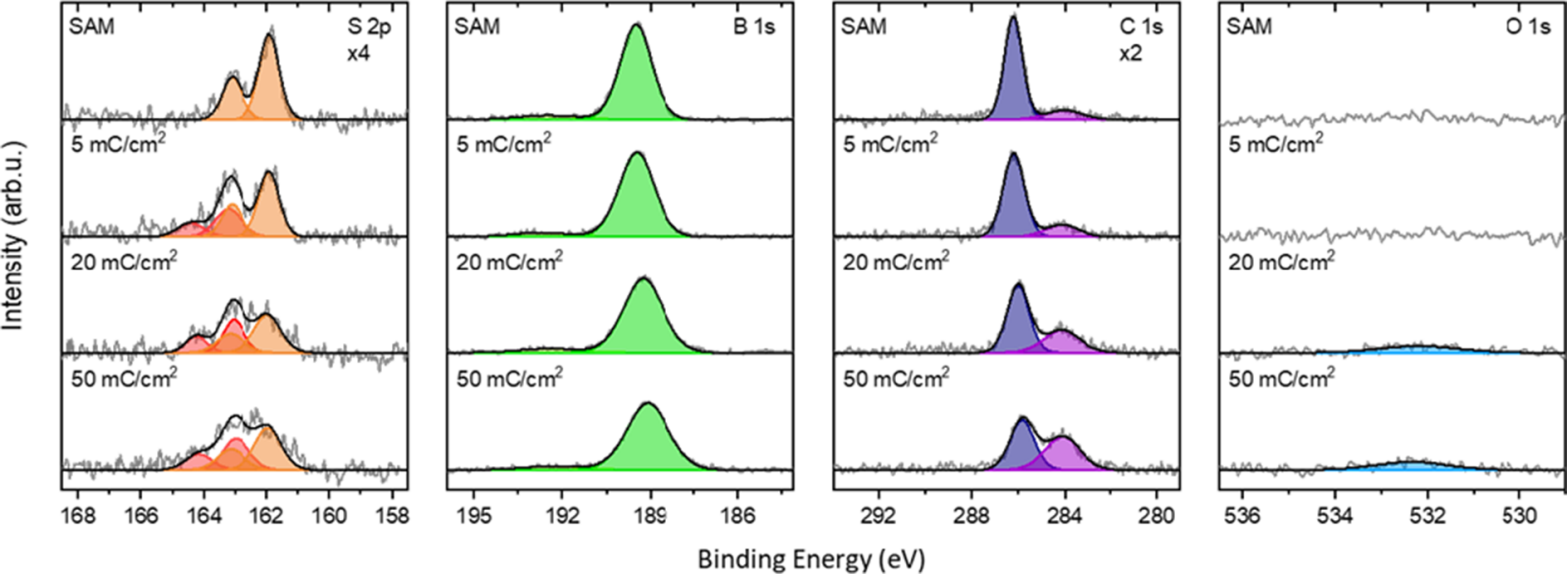
XP spectra of a ***mm*-SH** SAM on Ag(111) single crystal, stepwise cross-linked via electron irradiation with an energy of 50 eV. The spectra’s intensities have been multiplied by the indicated factor for better representation.

Next, the structural properties of the ***mm*** **-SH** SAM on Ag(111) were investigated by using STM and LEED. Figure 8A presents an STM image with even submolecular resolution, revealing an ordered arrangement of ***mm*** **-SH** molecules. The unit cell consisting one molecule is highlighted as a blue quadrilateral. Each molecule exhibits two distinct height maxima, which correspond to the two cages of the ***mm*** **-SH** molecular structure. The measured distance between both maxima is ∼0.5 nm, which is in agreement with the results shown in Figure 1. Based on the STM data, we also conclude that the molecules remain structurally intact after adsorption onto the Ag(111) surface.

**Figure 8:**
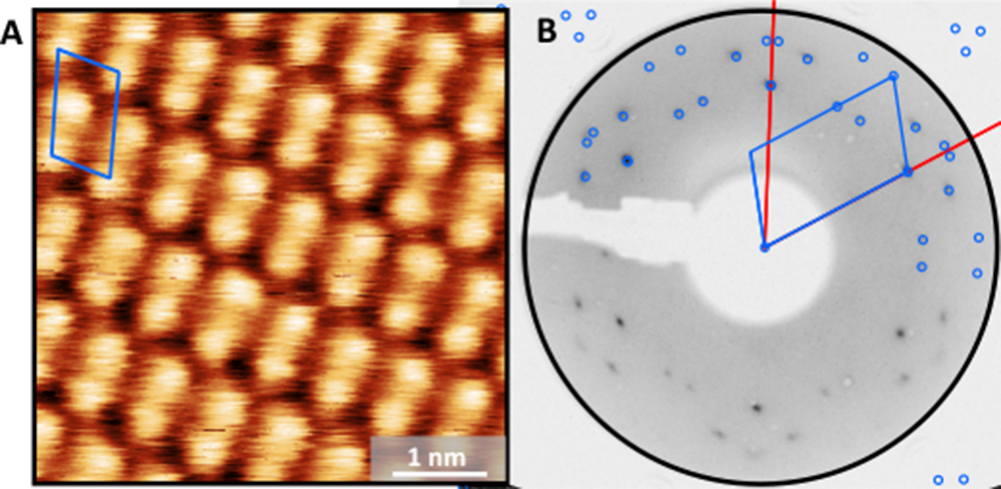
(A) STM image of the ***mm*** **-SH** SAM on Ag(111) (0.1 V, 1.0 nA, 293 K). (B) LEED pattern of ***mm*** **-SH** SAM on Ag(111) (25 eV, 293 K). The quadrilateral unit cell and the simulated LEED pattern of the ***mm*** **-SH** SAM are highlighted in blue. The red lines in the LEED pattern indicate the reciprocal lattice vectors of Ag(111).

To determine the lattice parameters of the ***mm*** **-SH** SAM on Ag(111) with high precision, quantitative LEED analysis was performed (Figure 8B). The identified oblique structure, which explains all visible LEED spots, is highlighted in blue. The measured lattice vectors |*a→*_1_| and |*a→*_2_| are 6.83 ± 0.07 Å and 11.47 ± 0.11 Å, respectively, with an enclosing angle of 109.46 ± 0.08 °. The lattice parameters and the epitaxy matrix obtained from the LEED analysis are summarized in Table S5 and show excellent agreement with the STM data. Within the uncertainties of the epitaxy matrix, the ***mm*** **-SH** lattice can be described by an online epitaxy.^54^ The structural fit to the LEED pattern includes three rotational domains that arise from the substrate symmetry. Furthermore, due to the presence of a glide plane, the first-order diffraction spots in the LEED image are systematically absent.^55,56^ This result shows that the ability of the ***mm*** **-SH** molecules to freely rotate does not contradict the formation of highly ordered SAMs on the Ag(111) surfaces. The combination of the thiolate bond to the substrate along with the high packing density of the molecules leads to a uniform orientation of the monolayer.

### Electron Irradiation-Induced Cross-Linking

After SAM formation, the carborane SAMs were stepwise converted into nanomembranes via low-energy electron irradiation-induced cross-linking. XPS was performed to investigate the structural changes induced by the electrons. These spectra are also shown in Figure 7. During cross-linking, new sulfur species at BEs of 163.0 eV (S 2p_3/2_) and 164.2 eV (S 2p_1/2_) is formed. This signal is typical for thiols and dithiols and it is an indicator that the thiolate bonds to the silver surface are lifted during electron irradiation while disulfide bridges between adjacent molecules are formed. This process effectively transforms the SAM into a laterally cross-linked network, consistent with previously reported electron-beam-induced restructuring of thiolate SAMs on metal surfaces- both for carborane based and purely organic systems.^57,58^ While the total amount of sulfur remains similar before and after irradiation, half of the sulfur attributed to thiolates at 161.9 eV (S 2p_3/2_) and 163.1 eV (S 2p_1/2_) is converted into the new species at higher binding energies.

After cross-linking, the B 1s signal broadens and its full width at half-maximum (FWHM) increases from 1.2 to 1.7 eV. The BE shifts by 0.4 eV toward lower binding energies to 189.1 eV. These changes are an indication for the formation of new boron species during the cross-linking process. Density functional theory (DFT) calculations on comparable carborane molecules suggest that this cross-linking occurs through the formation of new single B–B bonds, as well as bridging μ-B–H–B and μ-H bonds, leading to the creation of four-, five-, and six-membered rings that connect two or three molecules.^57^ The cross-linking also induces changes in the C 1s spectra. The peak assigned to the carborane atoms in the ***mm*** **-SH** SAM broadens, and its FWHM increases from 1.0 to 1.1 eV. Its BE shifts from 286.2 to 285.8 eV toward lower binding energies. The minor peak at 284.0 eV increases to ∼50% of the total carbon intensity. This indicates the formation of new types of bonds, including ones more comparable to the character of hydrocarbons. As the total amount of carbon remains unchanged during cross-linking, these new bonds are formed due to chemical changes rather than the deposition of additional carbon impurities. The effect of electron irradiation on the C 1s spectrum is consistent with that observed for B 1s, confirming that the SAM undergoes cross-linking during irradiation. The ratio of C:S:B after irradiation is found to be (2.6 ± 0.5):(0.7 ± 0.1):10. The elemental ratios as well as the effective thickness of 5 ± 1 Å remain therefore mainly unaffected during the cross-linking process. After electron irradiation, the LEED pattern is no longer observable, suggesting a lifting of the long-range order as the crystalline structure of the SAM is converted to a more amorphous nanomembrane. These findings are in line with our earlier work on other carborane SAMs,^57^ which showed that boron-centered reorganization is a general effect found in different carborane structures. In that way, carboranes mirror the properties of aryl and alkyl based SAM, which can also be converted into 2D nanomembranes upon electron irradiation.^58–60^ This suggests that the carborane cluster acts as a versatile cross-linking unit, enabling the controlled conversion of ordered SAMs into stable nanomembranes without significant material loss or contamination.

### Formation of Free-Standing Membranes

Afterward, SAMs of ***mm*** **-SH** were also prepared from solution under inert conditions on a Au/mica substrate. After cross-linking using low-energy electrons with an energy of 50 eV and an electron dose of 50 mC/cm^2^, these membranes were transferred on SiO_2_/Si substrates and transmission electron microscope (TEM) grids using a PMMA-assisted transfer method.^61^ In the optical microscopy image (Figure 9A), the transferred membrane (blue) is visible on the silicon oxide wafer due to interference effects, confirming the successful transfer. However, the SEM image reveals a tangle-web-like structure when prepared freestanding (Figure 9B). The electron-induced cross-linking of this relatively more complicated molecular architecture with a greater degree of flexibility (i.e., with two axes of practically free rotation) compared to previously published carborane SAMs,^51,57^ and also exhibiting lower density of the surface coverage, leads to the formation of 2D nanomembranes resembling molecular tangle-webs without any periodicity or regular pattern, but compact enough to be mechanically detached from the metal substrate and transported onto another substrate.

**Figure 9:**
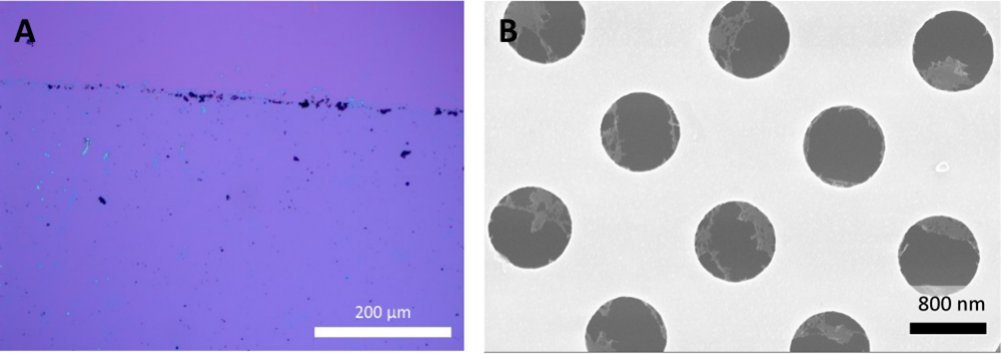
(A) Optical microscopy image of a transferred *mm*-SH-based membrane on a Si/SiO_2_ wafer. (B) SEM image of the membrane after transfer to a TEM grid.

## Conclusions

In conclusion, we have successfully synthesized and characterized bis-*meta*-carborane-thiol (***mm*** **-SH**), a rod-like molecule with distinct rotational flexibility and intramolecular dipole–dipole interactions. The supramolecular crystal structure, driven by the chain-like interactions between thiol groups and BH vertices, exhibits a layered structure with 2D network with unique hydrogen-bonding interactions. Free rotation around two molecular axes, combined with the low energy barriers between rotamers, enables unusually flexible, yet stable, supramolecular packing. Notably, this rotation results in rotamers with dipole moments ranging in value from 0.64 to 3.78 D, and marking ***mm*** **-SH** as the first cluster SAMs constituent with conformationally dependent dipole behavior. Importantly, this rotational motion persists at both room temperature and at the lowest accessible temperature (95K) as determined by SCXRD. We are currently investigating the possibility of freezing this rotational behavior at even lower temperatures.

We also demonstrate how the unique structural complexity and flexibility of the ***mm-SH*** carborane molecule influence the formation and properties of SAMs on metal surfaces. SAMs produced by PVD display highly ordered structures, while solution-phase deposition yields less dense, more disordered monolayers. Comprehensive XPS, STM, and LEED analyses confirm the integrity and composition of the monolayers and elucidate the chemical changes induced by their electron irradiation induced cross-linking, which converts these monolayers into mechanically stable, 2D nanomembranes with tangle-web morphology.

Extending carborane arrays beyond the dimer could yield dynamic architecture with multiple rotating units, opening avenues for tunable molecular rotors and adaptive materials. Altogether, this study enhances our understanding of the structure–property relationship in carborane-based SAMs, suggesting potential applications in molecular electronics and nanotechnology, where tunable dipole interactions are desired.

## Supplementary Material









## Notes

Complete information on the NMR spectra (both experimental and computational), IR spectra, ESI mass spectra, results from thermal differential analysis, tables with structural data for X-ray diffraction determined, X-ray photoelectron spectra of the respective SAM, and further computational data, and cif files included (PDF)
Optimized structure ***m1*** (XYZ)
Optimized structure ***mm*** (XYZ)
Optimized structure ***mm-SH*** (XYZ)
