# Supplementary material for: Thiolated Bis-meta-Carborane: A Molecular Rotor with Conformation-Dependent Dipole Moment
Source: JACS Au. 2026 Jan 27;6(2):1327–36. doi: 10.1021/jacsau.5c01656 (PMC12933341; doi:10.1021/jacsau.5c01656)
Supplement: Supplementary file 1 [file au5c01656_si_001.pdf]

Supporting Information

Thiolated bis-*meta*-Carborane: A Molecular Rotor with Conformation-Dependent Dipole Moment

Deepak Kumar Patel [a],[b]‡, Martha Frey [c]‡, Monika Kučeráková [d]*, Jan Macháček [a], Dmytro Bavol [a], Julian Picker [c], Christof Neumann [c], Zdeněk Bastl [e], Michal Dušek [d], Sundargopal Ghosh [b]*, Andrey Turchanin [c]*, Thalappil Pradeep [b]*, and Tomas Base [a]*

[a] Institute of Inorganic Chemistry, The Czech Academy of Science, 25068 Rez, Czech Republic. E-mail: tbase@iic.cas.cz

[b] DST Unit of Nanoscience (DST UNS) and Thematic Unit of Excellence (TUE), Department of Chemistry, Indian Institute of Technology, Madras, Chennai-600036, India. E-mail: pradeep@iitm.ac.in , sghosh@iitm.ac.in

[c] Institute of Physical Chemistry Friedrich Schiller University Jena, 07743 Jena, Germany E-mail: andrey.turchanin@uni-jena.de

[d] Institute of Physics, The Czech Academy of Science, 182 21 Prague 8, Czech Republic. E-mail: kucerakova@fzu.cz

[e] J. Heyrovský Institute of Physical Chemistry, The Czech Academy of Sciences, Dolejškova 2155/3, 18200 Prague 8, Czech Republic

‡ These authors contributed equally to this work

Supporting Information Content

1. Experimental Section

2. Schematic of the synthesis

3. Schematic representations of the molecular structures (***mm*-SH**, ***mm***, ***m*1**, ***m***) for comparison of selected distances

4. Nuclear Magnetic Resonance (NMR) spectra

5. Mass Spectra (MS)

6. Infrared Spectrum (IR)

7. Thermogravimetric analysis / Differential Thermal Gravimetry (TGA/DTG)

8. X-ray Diffraction Analysis (Supramolecular packing - stacking of 2D sheets,

9. Dipole moments of conformers and the transition states

10. X-ray Photoelectron Spectroscopy Analysis

11. Low-Energy Electron Diffraction Analysis

1. Experimental Section

General procedure and instrumentation

All the experiments were performed under an inert (Argon) atmosphere using the standard Schlenk-line vacuum technique. Prior to use, all the solvents (purchased form LACH-NER, s.r.o., Czech Republic) were dried with sodium in the presence of benzophenone until dark blue or purple color, and distilled off freshly before use. Chloroform-d was purchased from ACROS organic (Thermo Scientific Chemicals)**.** Starting *meta*-carborane (1,7-$C_2B_{10}H_{12}$) was purchased from Katchem s.r.o., Czech Republic, and used as received. Other chemicals, such as sulfur or *n*-BuLi (2.5 M solution in hexanes) were purchased from Aldrich, and used as received.

Compound Synthesis: 1-(*meta*-carboranyl)-*meta*-carborane-7-thiol (***mm*-SH**)

Under the inert atmosphere of Ar, 1-(1'-*meta*-carboranyl)-*meta*-carborane, ***mm***, (1.42 g) was dissolved in approximately 250 mL of dry (refluxed over pieces of metal Na in the presence of benzophenone until dark blue/purple in color) and freshly distilled Et_2O. The solution was cooled in ice-water bath for 50 min, and a solution of *n*-BuLi (2.18 mL of 2.5 M solution in hexanes) was dropwise added through septum. The solution turned white cloudy due to the Li salt formation. The ice-water bath was removed, and the solution was stirred for another 40 minutes allowing it to warm up to room temperature. Then, the mixture was cooled by placing the flask with the mixture in ice-water bath again, and 5 minutes later, sulfur powder (157 mg) was added all at once. After one hour of vigorous stirring, all sulfur practically dissolved, the ice-water bath was removed, and the reaction was quenched by adding distilled water (200 mL). The two-phase mixture was stirred at room temperature for 10 minutes, and the organic (Et_2O) part (containing unreacted ***mm***) was separated to recover the starting material. The aqueous phase was acidified by adding an aqueous solution of HCl (100 mL, 1M) which caused precipitation of the desired white solid product. This crude product was then extracted by Et_2O (4 × 50 mL, used as received), and combined Et_2O fractions were dried by standing above anhydrous $MgSO_4$ overnight. The mixture was then filtered, and the solvent was evaporated under reduced pressure on a rotary evaporator to yield 0.8 g of NMR pure product, ***mm*-SH**.

MS (ESI+): m/z calculated for $(C_2B_{10}H_{11})_2S$ (***mm*-SH)** monocationic species: 318.34 experimentally found 318.50.

$^{11}B\{^1H\}$ NMR (192.6 MHz, 295 K, CDCl3): δ-4.13 ppm (B12, B5') > -7.85 ppm (B5) > -8.63 (B12') > -10.44 (B9, B10, B4', B6', B9', and B10') > -11.32 (B4, B6) > -11.66 (B8, B11) > -12.35 (B2, B3) > -14.70 (B8', B11') > -15.53 (B2', B3').

Crystal Data for ***mm*-SH:** Empirical formula = $C_4B_{20}H_{22}S$, Mr = 317.5, Tetragonal, space group P42/mnm, a = 11.488(2) Å, b = 11.488(2) Å, c = 6.882(2) Å, α = 90°, β = 90°, γ = 90°, V = 908.2(3) Å3, Z = 2, ρcalc. = 1.161g/cm3, μ = 1.36 mm–1, F(000) = 322, R1 = 0.1457, wR2 = 2659 independent reflections and 527 parameters (for more detail, see Table S2).

NMR

NMR spectra were recorded on a 600 MHz JEOL NMR spectrometer. Chemical shifts were analyzed with reference to the solvent (δ=7.26 ppm, $CDCl_3$, 600 MHz, 295K) and boron (δ=0 ppm, relative to $BF_3(OEt)_2$) in $CDCl_3$, 192.6 MHz, 295K) for the ^1H, and $^{11}B\{^1H\}$ NMR spectra, respectively. Complete assignment with all the spectra are provided in the Supporting information.

GC MS

GC-MS was performed on a TRACE™ 1310 gas chromatograph interfaced to an ISQ™ 7000 Single Quadrupole mass spectrometer. Chromatographic conditions were as follows: column TraceGOLD (30 m × 0.25 mm *I.D.*, 0.25 µm film thickness) of phase 5% diphenyl – 95% dimethylpolysiloxane. The oven program started at an initial temperature of 50 °C for 1 min which was increased at a rate of 25 °C/min to 280 °C and finally held at 280 °C for 3 min. The GC injectors were equipped with a 3.4 mm *I.D.* and were maintained at 270 °C with a 1:75 split ratio; the injection volume was 1 µL. The carrier gas was helium (99.996%) at a 1.5 mL/min flow rate. Ionisation was performed by electron impact (EI) mode at 70 eV. The temperatures used were 270 °C for the MS Transfer Line and 280 °C for the MS Ion Source; the mass range from 50 to 450 amu.

IR spectra

Infrared spectrum of mm-SH was measured in a KBr pellet (1.2 mg of the sample / 300 mg KBr) in the region from 4 000 cm^{-1} to 400 cm^{-1} (with a resolution of 4 cm^{-1}) using Nicolet Nexus 670 FTIR spectrometer, Thermo Electron Corporation.

DTG/TGA

The thermal analysis of the samples was performed using Setsys Evolution 1750 (Setaram) equipped with MS detector QMG 422 (Pfeiffer).

Computational study

Quantum chemistry calculations were performed by the NWChem1[55] package. The geometries were optimised [56] by the means of the density functional theory with the hybrid exchange–correlation functional PBE0 [57, 58] using Jensen's double–zeta segmented polarisation consistent basis set pcseg-1.[59] A series of optimisations was performed in internal coordinates with the torsion angle among the cage carbon atoms (7, 1, 1', 7') kept fixed; then the angle was relaxed, and a full optimisation to a local minimum or, for the high energy conformations found, to a saddle point, conducted without constraints. The starting values of the torsion angle were mainly concentrated around 0, 36, 72, 108, 144, and 180 degrees, reflecting the pentagonal arangement around the C–C link between the cages, and the mirror symmetry of the molecule.

Single-crystal X-ray diffraction analysis

Single-crystals suitable for X-ray diffraction analysis were grown in a PTFE-closed glass vial placed on a warm oven (temperature of the oven surface was 35-40 °C). The X-ray diffraction data of the single-crystals were collected with Rigaku OD Supernova using Atlas S2 CCD detector and mirror collimated Cu-Kα (λ = 1.54184 Å) from a micro-focused sealed X-ray tube. The samples were at measured at 100 K (cooling rate of 5 K/min) as well as at room temperature (300 K). Integration of the CCD images, absorption correction, and scaling were done by the program CrysAlisPro 1.171.41.123a (Rigaku Oxford Diffraction, 2022). Crystal structures were solved by charge flipping with program SUPERFLIP [60] and refined with the Jana2020 program package [61] by full-matrix least-squares technique on F^2. The crystal data collection and analysis details are reported in more detail in the supporting information.

Crystal data for ***mm*-SH** (100 K):

Empirical formula = $C_4B_{20}H_{22}S_1$, M_r = 317.5, tetragonal, Space group P42/mnm, a= 11.488(2) Å, b=11.488(2) Å, c=6.882(2) Å, α=90°, β=90°, γ=90°, V=908.2(3) $Å^3$, Z=2, $\rho_{calc.}$=1.161 g/cm^3, μ=1.36 mm^{-1}, F(000)= 322, R1=0.1137, wR2= 0.2690, 547 Independent reflections and 31 parameters.

Formation of Self-Assembled Monolayers (SAMs)

Preaparation via physical vapor deposition

The SAMs were prepared by evaporation in the same UHV system (< 2 $\times 10^{-10}$ mbar) used for XPS, UPS, STM, and LEED measurements using a molecular evaporator (Kentax). The ***mm*-SH** molecules were evaporated at 80 °C for 1 h on 300 nm Ag/ mica substrates (Georg Albert PVD) or silver single crystal (Ag (111), Matek, purity 99,999 %) respectively, which were held at room temperature. The substrates were cleaned before by repeated sputtering with Ar^+ (1keV, 10 mA) and annealing at 370 °C.

Preparation from solution

The SAMs of ***mm*-SH** were prepared by immersing freshly-cleaned surfaces of 300 nm gold film deposited on mica (Georg Albert PVD) to a dichloromethane (DCM) solution of the starting derivative (10.1 mg of ***mm*-SH** dissolved in 17 ml of the solvent) at room temperature for 22 h under nitrogen. SAM-modified surfaces were then consecutively washed with pure DCM and EtOH, and dried under a stream of Ar before inserting them in the XP spectrometer. Prior to preparation, gold substrates were cleaned with oxygen plasma, rinsed with EtOH and dried under a stream of N_2. All glassware for used for the SAM preparation was cleaned with piranha solution, rinsed with ultrapure water (0.056 μS/cm, MembraPure Aquinity2 E35) and dried.

Nanomembrane preparation

The electron irradiation took place in the same UHV system used for the XPS measurements. The samples were irradiated at 50 eV at an electron dose of 50 mC/ cm^2 using a NEK 150SC electron gun (Staib).

After irradiation, the nanomembranes were transferred onto silicon wafers with an oxide surface layer (300 nm, Sil'tronix) and onto TEM grids (Quantifoil R 0.6/1). To transfer the nanomembrane, a 70 nm thick polymethyl methacrylate (PMMA) layer was applied to the surface. For this purpose, PMMA (50K, AR-P 631.04) was spin-coated onto the nanomembrane while it was still on the gold substrate and heated for 5 min at 50 °C on a hotplate. The second layer of PMMA (950K, AR-P 671.04) was then applied under the same conditions and baked for 30 min at 50 °C.

To separate the gold substrate from the nanomembrane, the sample was placed on the surface of an iodine-potassium iodide solution (I_2/ KI/ H_2O in a mass ratio of 1:4:10) for 30 minutes. After separation, excess iodine was reduced with a sodium thiosulphate solution. The samples were then repeatedly washed with ultrapure water (0.056 µS/ cm, MembraPure Aquinity2 E35), transferred onto the silicon wafer or TEM mesh and dried for 4 h at 50 °C. To remove the PMMA layer after transfer, the samples on the silicon wafers were immersed in acetone for 20 min, rinsed with isopropanol and dried under a stream of nitrogen. To minimize any damage to the free-standing membranes, supercritical drying (Autosamdri-815, Tousimis) was performed for the samples on TEM meshes.

The solvents; 2-propanol (H2O ≤ 0.1 %, purity ≥ 99.8 %), acetone (H2O ≤ 0.2%, purity ≥ 99.8%), and ethanol (H2O ≤ 0.2%, purity ≥ 99.8%); were all obtained from VWR Chemicals and used as delivered.

X-ray Photoelectron Spectroscopy (crystalline ***mm*-SH**)

The XPS of the samples were measured using a modified ESCA 3 MkII multi-technique spectrometer equipped with a hemispherical electron analyzer operated in fixed transmission mode. Al K_α radiation was used for electron excitation. The binding energy scale was calibrated by using the Au $4f_{7/2}$ (84.0 eV) and Cu $2p_{3/2}$ (932.6 eV) photoemission lines. The pressure in the XPS analysis chamber during spectral acquisition was 6×10^{-9} mbar. The powdered sample was spread on an aluminum surface. The high-resolution spectra were collected at 20 eV pass energy and a takeoff angle of 45° with respect to the macroscopic surface normal. Survey scan spectra and high-resolution spectra of B 1s, C 1s, and S 2p photoelectrons were measured. The spectra were fit after subtraction of Shirley background using the Gaussian−Lorentzian line shape and nonlinear least-squares algorithms (CasaXPS version 2.3.15. software). The binding energies of the powdered samples were referenced to the C 1s peak of adventitious carbon at 284.8 eV. Quantification of the elemental concentrations was accomplished by correcting the photoelectron peak intensities for their cross-sections and for the analyzer transmission function. In calculations, homogeneous compositions of the analyzed samples layer were assumed.

X-ray Photoelectron Spectroscopy (SAMs and cross-linked samples)

XPS was measured using a UHV Multiprobe system (Scienta Omicron) with a monochromatic X-ray source (Al K_α, 1486.7 eV). The electron analyser (Argus CU) has a spectral energy resolution of 0.6 eV (XPS). The pass energies of 50 eV for the survey XP spectra, and 30 eV for high-resolution XP spectra were used. The XP spectra were calibrated referencing the binding energy of Ag $3d_{5/2}$ at 368.2 eV and fitted using Voigt functions (30:70) after linear background subtraction. Calculations of stoichiometry were performed with the software CasaXPS using the relative sensitivity factors of 1.68 (S 2p), 0.49 (B 1s), and 1.00 (C 1s); layer thickness was calculated using the Beer-Lambert equation. A mean free path of 27 Å was used for electrons that were released from the silver substrate and reached the detector through the SAM.[62][63] The analyser's acceptance angle (α) was 19°.

Low-Energy Electron Diffraction (LEED)

LEED measurements were performed with a single microchannel plate (SMCP) LEED setup (Scienta Omicron) at room temperature. The beam energies were varied between 10 and 120 eV. The LEED patterns were corrected from distortions using the LEEDCal software.[64] For the quantitative LEED analysis, the distortion-free LEED patterns were analyzed by fitting the visible LEED spots to a structural model using the LEEDLab software.[65] As a reference, the hexagonal lattice of Ag(111) with a lattice constant of 2.8894 Å was utilized.[66]

Scanning Tunneling Microscopy (STM)

STM measurements were employed using a VT SPM (Scienta Omicron) in UHV at room temperature. A W tip, which was sputtered with Ar^+ ions prior to measurements, was used. Typical parameters for the bias voltage and the tunneling current were 0.1 V and 1.0 nA, respectively. STM images were processed and analyzed using the Gwyddion software.[67] The STM images were corrected from thermal drift using the lattice parameters obtained from the LEED analysis.

Optical microscopy

Optical microscopy was performed in brightfield mode with a Zeiss Axio Imager Z1.m microscope. The microscope was equipped with a thermoelectrically cooled 3-megapixel CCD camera (Axio-cam 503 color).

Scanning Electron Microscopy (SEM)

The SEM images were obtained with a Zeiss Sigma VP field emission scanning electron microscope at a beam power of 10 kV using the system's in-lens detector.

2. Schematic of the Synthesis

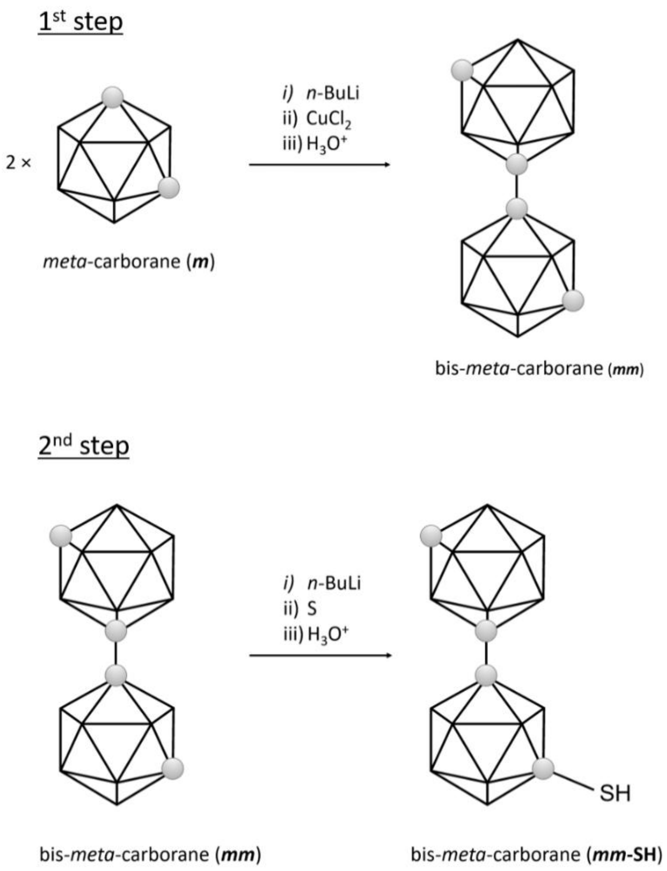

Figure S1. Schematic illustration of synthesis of ***mm*-SH**.

3. Schematic representation of the molecular structures (*mm*-SH, *mm*, *m*1, *m*) for comparison of selected distances

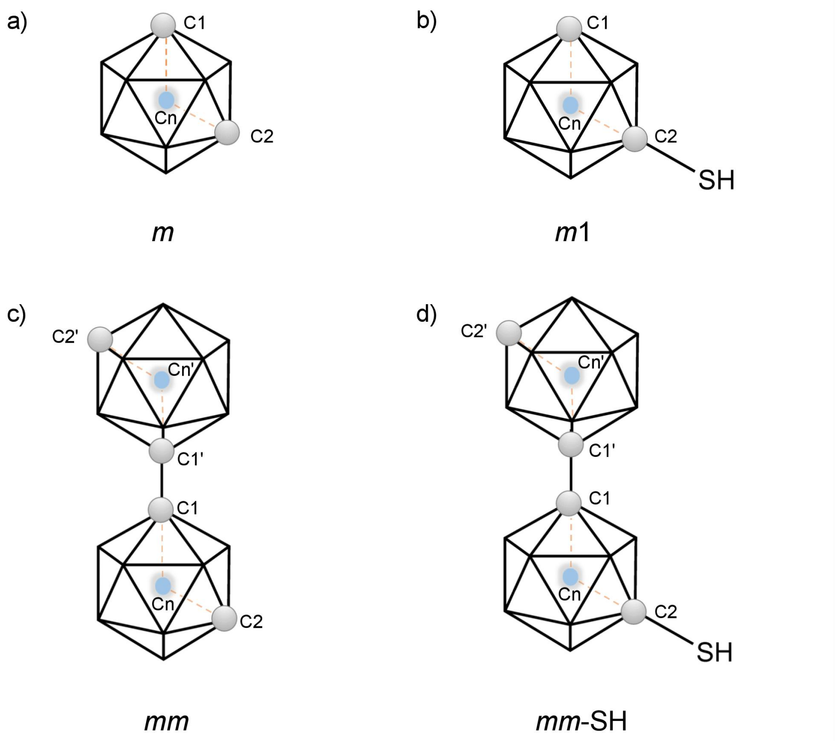

Figure S2. Schematic illustration of selected centroid-vertex distances. Centroids: Cn and Cn', Carbon vertices: C1, C2, C1', C2'.

4. NMR

Table S1: Measured ^{11}B, ^{1}H NMR chemical shift data for ***mm*-SH** in $CDCl_3$ solution at 300 K.

	***mm*-SH**	
Assign.	δ(^{11}B)	δ(^{1}H)
1	-	-
2,3	-12.35	
4,6	-11.32	
5	-7.85	
7	-	2.96 *(S*H*)
8,11	-11.66	
9,10	-10.44	
12	-4.13	
1’	-	-
2’,3’	-15.53	
4’,6’	-10.44	
5’	-4.13	1.55 C*H*
7’	-	
8’,11’	-14.7	
9’,10’	-10.44	
12’	-8.63	

*(S*H*) hydrogen atom from the S*H* group

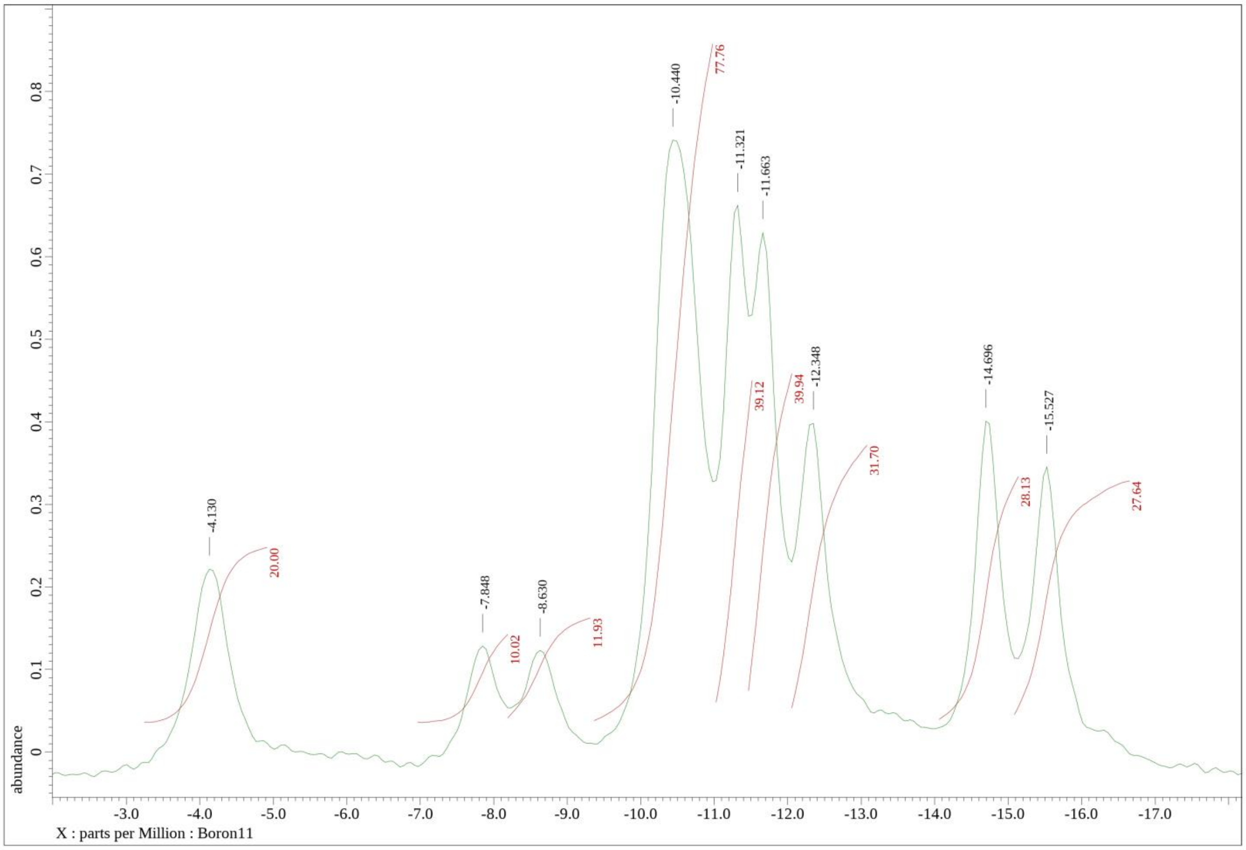

Figure S3. Experimental $^{11}B\{^{1}H\}$ decoupled spectra of ***mm*-SH**.

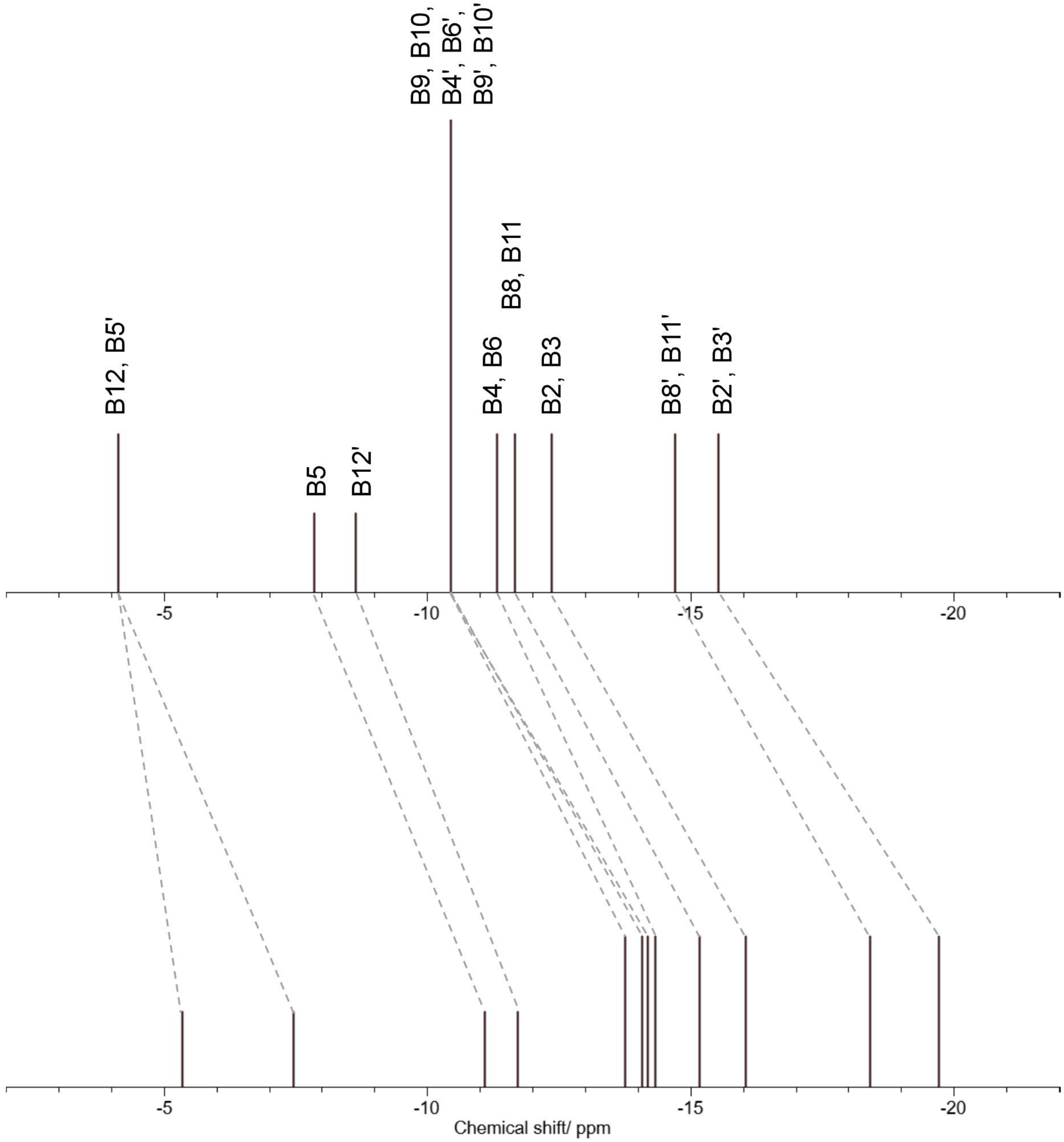

Figure S4. Correlation between the (top) experimental and (bottom) computational ^{11}B NMR spectra of ***mm*-SH**. Ratio of the boron signal intensity of experimental ^{11}B NMR: 2:1:1:6:2:2:2:2:2

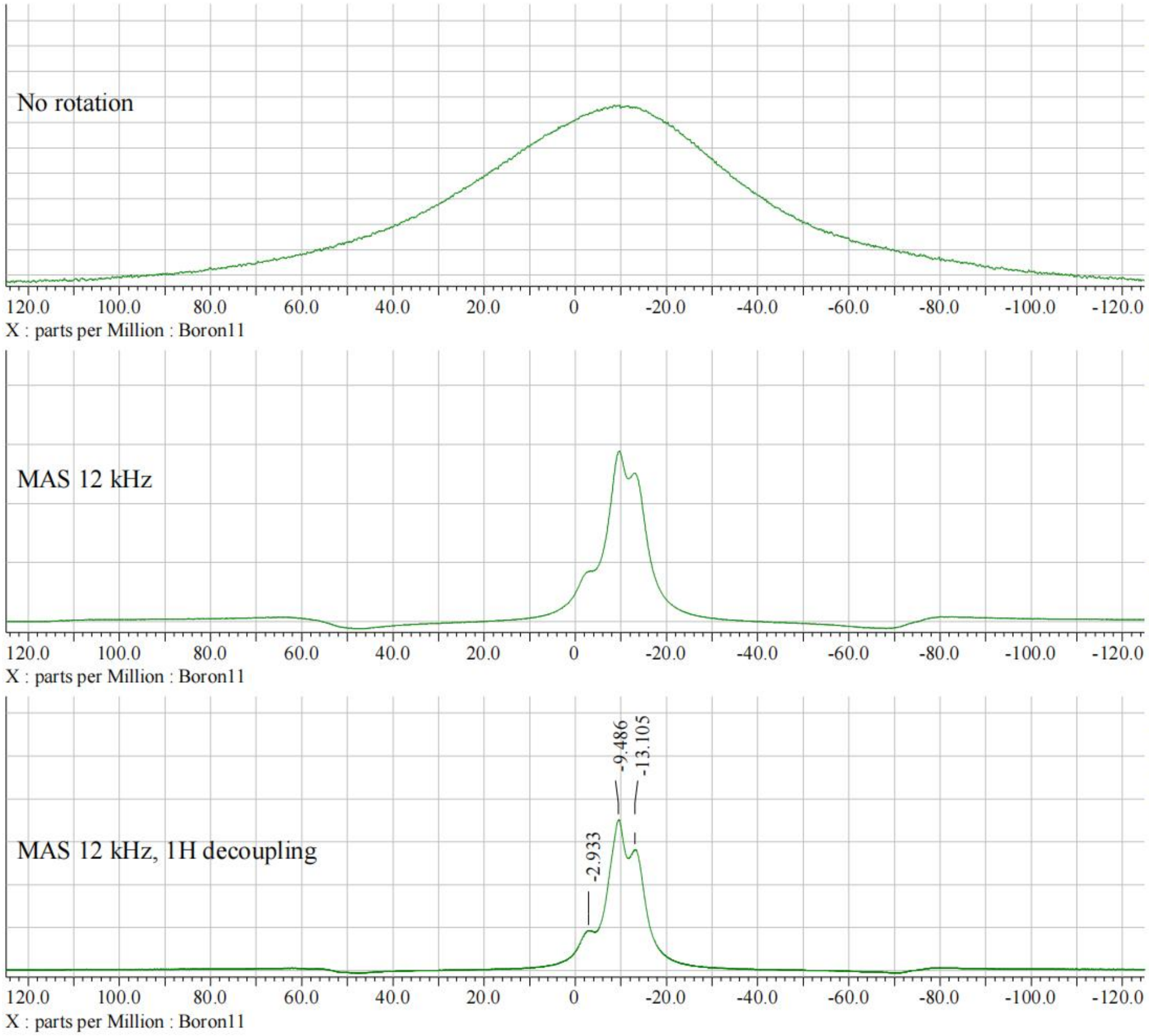

Figure S5. Experimental ^{11}B spectra of ***mm*** in solid state at 300K.

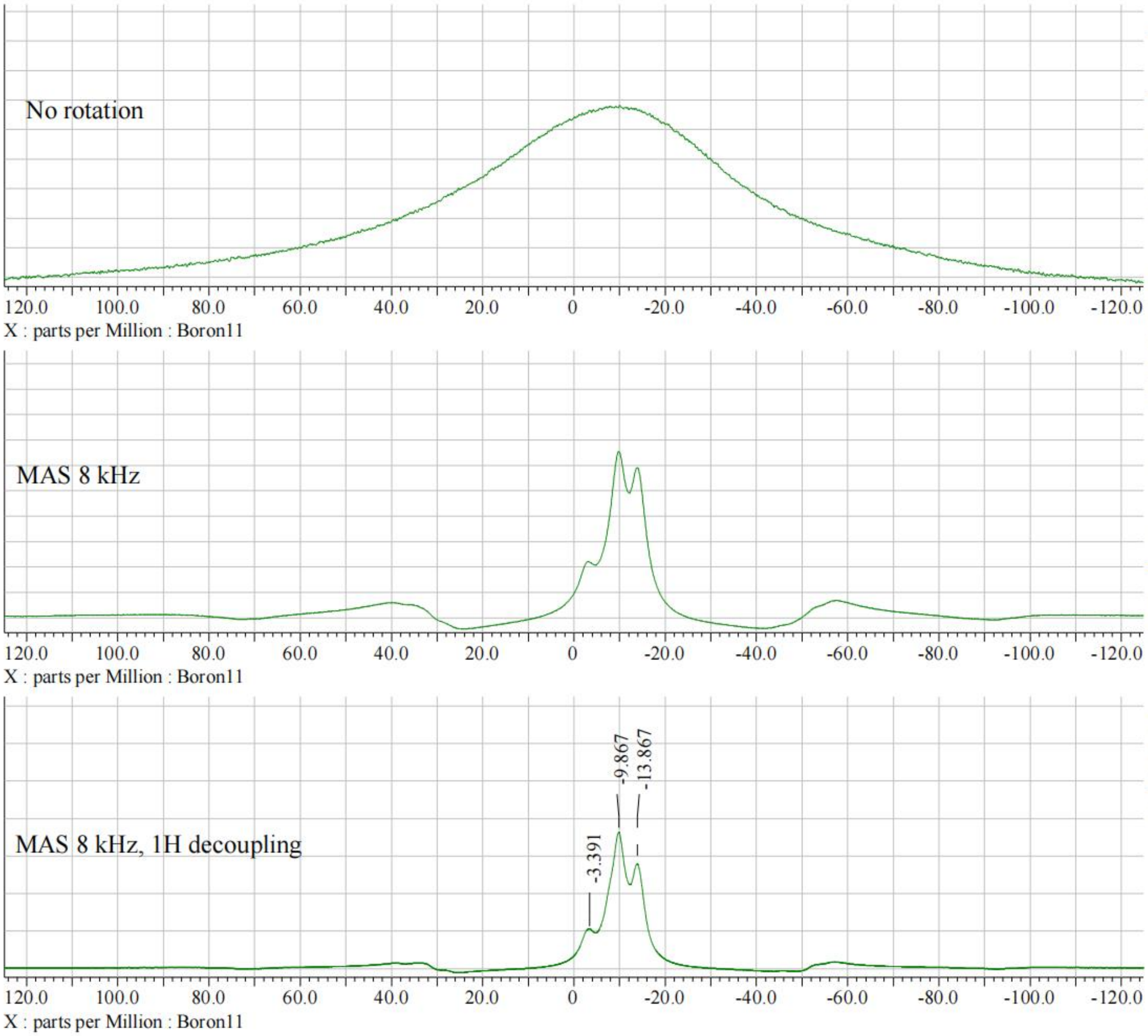

Figure S6. Experimental ^{11}B spectra of ***mm*-SH** in solid state at 300K.

5. MS

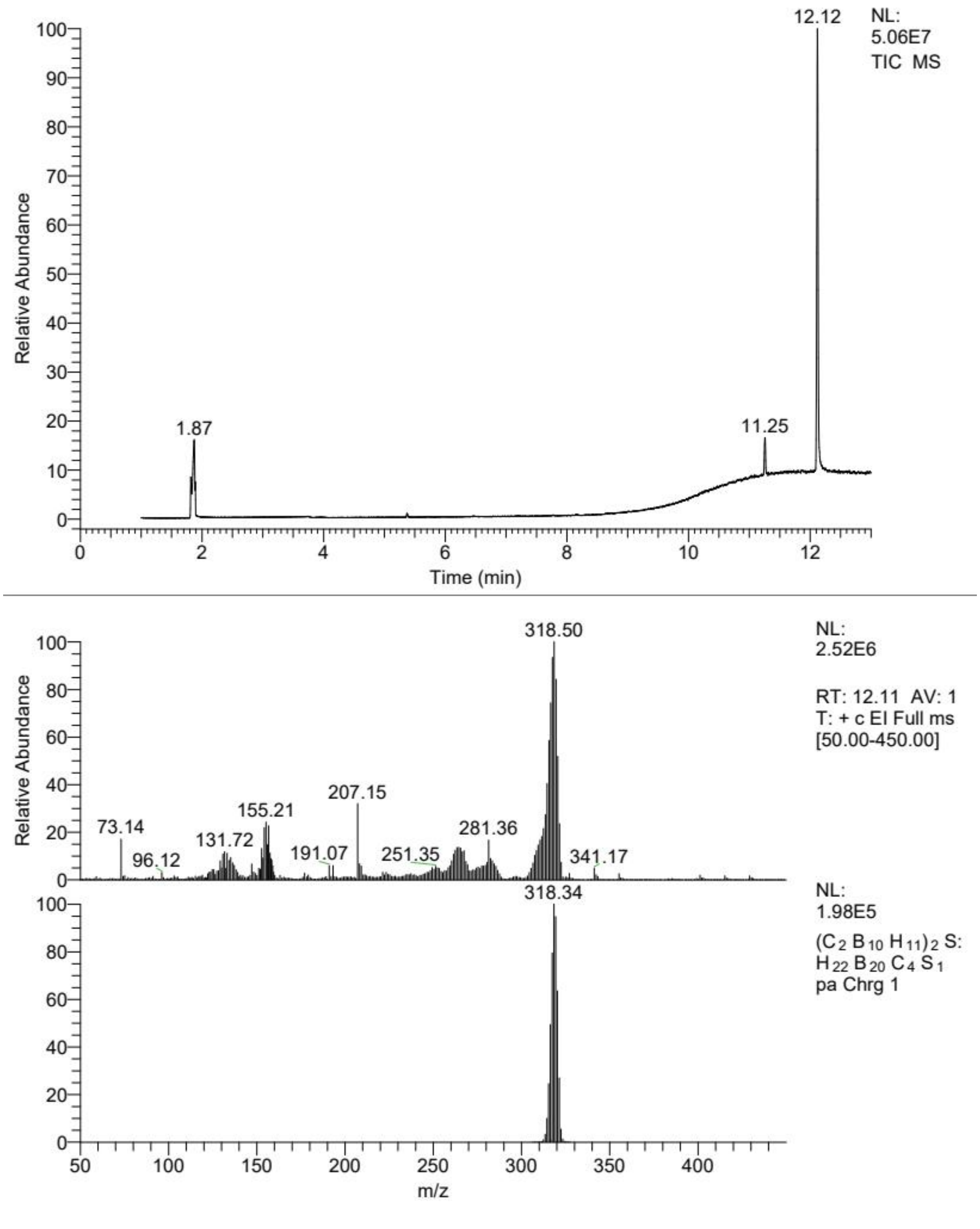

Figure S7. Gas Chromatography with Mass Spectra of ***mm*-SH**.

6. IR spectrum of *mm*-SH

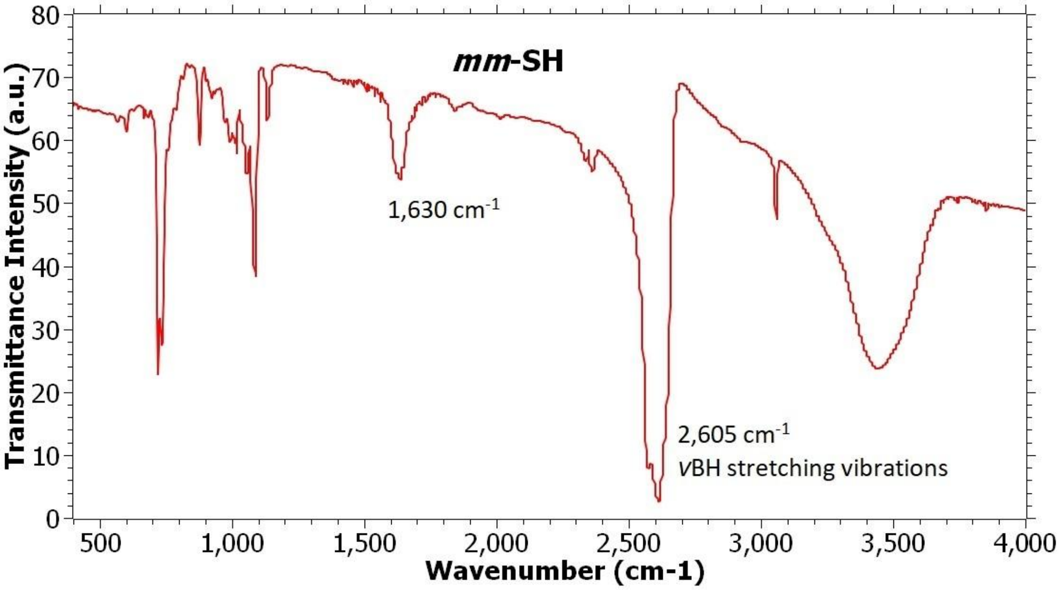

Figure S8. IR spectrum of ***mm*-SH**.in the region from 4,000 cm^{-1} to 400 cm^{-1}.

7. Thermogravimetric Analysis

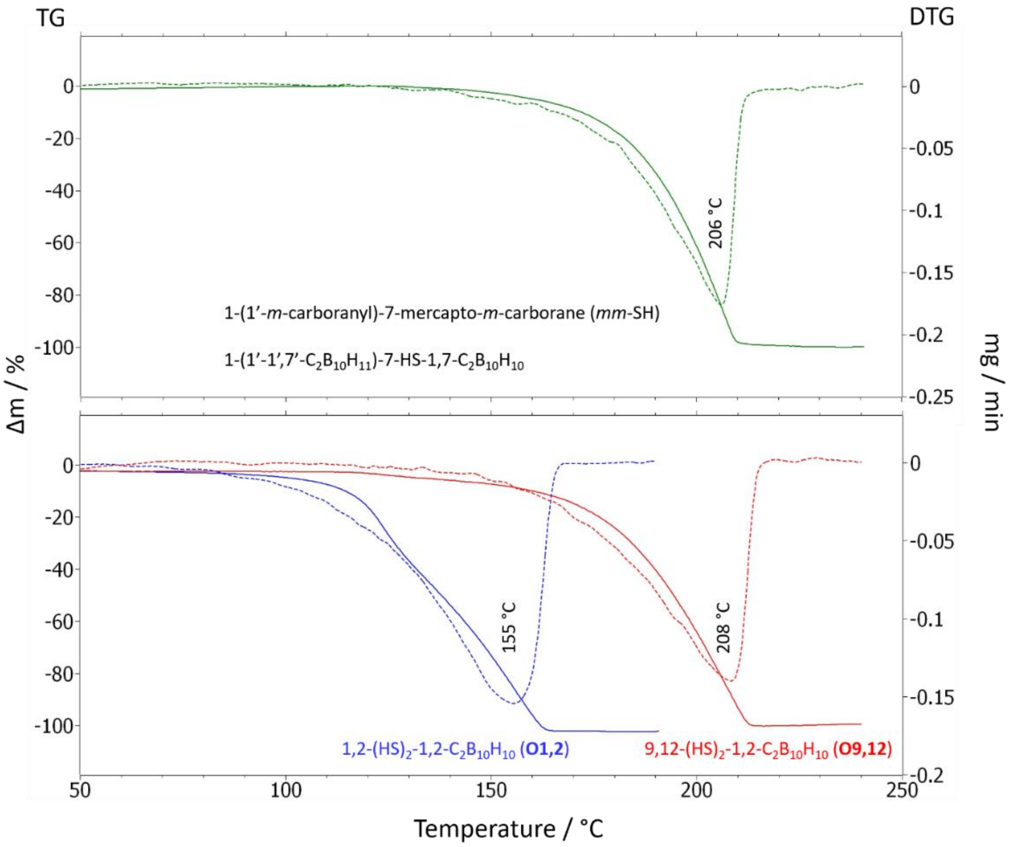

Figure S9. DT analysis showing the volatility of ***mm*-SH** in comparison with two previously studied dithol derivatives.

8. X-ray diffraction Analysis

Table S2. Crystallographic collection and refinement data for ***mm*-SH** measured at 100 K.

	***mm*-SH** – at 100 K
CCDC	2426544
Empirical formula	$C_4B_{20}H_{22}S$
Diffractometer	four-cycle diffractometer<br>Cryostream, AtlasS2, Rigaku OD Supernova

	***mm*-SH** – at 100 K
M_r /g mol^{-1}	317.5 (formula weight)
T/K	100
Wavelength / Å	1.54184
Crystal system	Tetragonal
Space group	P42/mnm
a/Å	11.488(2)
b/Å	11.488(2)
c/Å	6.882(2)
α/deg	90
β/deg	90
γ/deg	90
V/Å^3	908.2(3)
Z	2
Calc. density/g cm^{-3}	1.161
μ mm^{-1}	1.36
F(000)	322
Crystal size /mm^3	0.16 × 0.06 × 0.05
θ range /°	5.4–72.6°
Index ranges /*hkl*	-14-11, -14-14, -7-8
Reflections collected (R_{int})	0.065
Independent reflections	527
Completeness /% to θ /°	99, 73.86
Absorption correction	Multi-scan
Max. and min. transmission	Tmin = 0.581, Tmax = 1
Data / restraints / constraints / parameters	527/0/27/31
Goodness-of-fit on F^2	2.82

	***mm*-SH** – at 100 K
*R*1, *wR*2 [I>3σ(I)]	0.1011, 0.2459
*R*1, *wR*2 (all data)	0.1457, 0.2659
Largest diff. peak and hole, eÅ^{-3}	0.33, −0.26

Table S3. Crystallographic collection and refinement data for ***mm*-SH** measured at 300 K.

	***mm*-SH** – at 300 K
CCDC	2426543
Empirical formula	$C_4B_{20}H_{22}S$
Diffractometer	four-cycle diffractometer<br>Cryostream, AtlasS2, Rigaku OD Supernova
M_r /g mol^{-1}	317.5 (formula weight)
T/K	300
Wavelength / Å	1.54184
Crystal system	Tetragonal
Space group	P42/mnm
a/Å	11.6005 (9)
b/Å	11.6005 (9)
c/Å	6.9688 (6)
α/deg	90
β/deg	90
γ/deg	90
V/Å^3	937.80(13)
Z	2
Calc. density/g cm^{-3}	1.124

	***mm*-SH** – at 300 K
μ mm^{-1}	1.32
F(000)	322
Crystal size /mm^3	0.16 × 0.06 × 0.05
θ range /°	5.4–73.8°
Index ranges /*hkl*	-11-14, -12-14, -8-7
Reflections collected (R_{int})	0.056
Independent reflections	547
Completeness /% to θ /°	99, 73.78
Absorption correction	Multi-scan
Max. and min. transmission	Tmin = 0.477, Tmax = 1
Data / restraints / constraints / parameters	547/0/27/31
Goodness-of-fit on F^2	2.78
*R*1, *wR*2 [I>3σ(I)]	0.1137, 0.2690
*R*1, *wR*2 (all data)	0.1752, 0.2997
Largest diff. peak and hole, $eÅ^{-3}$	0.17, −0.24

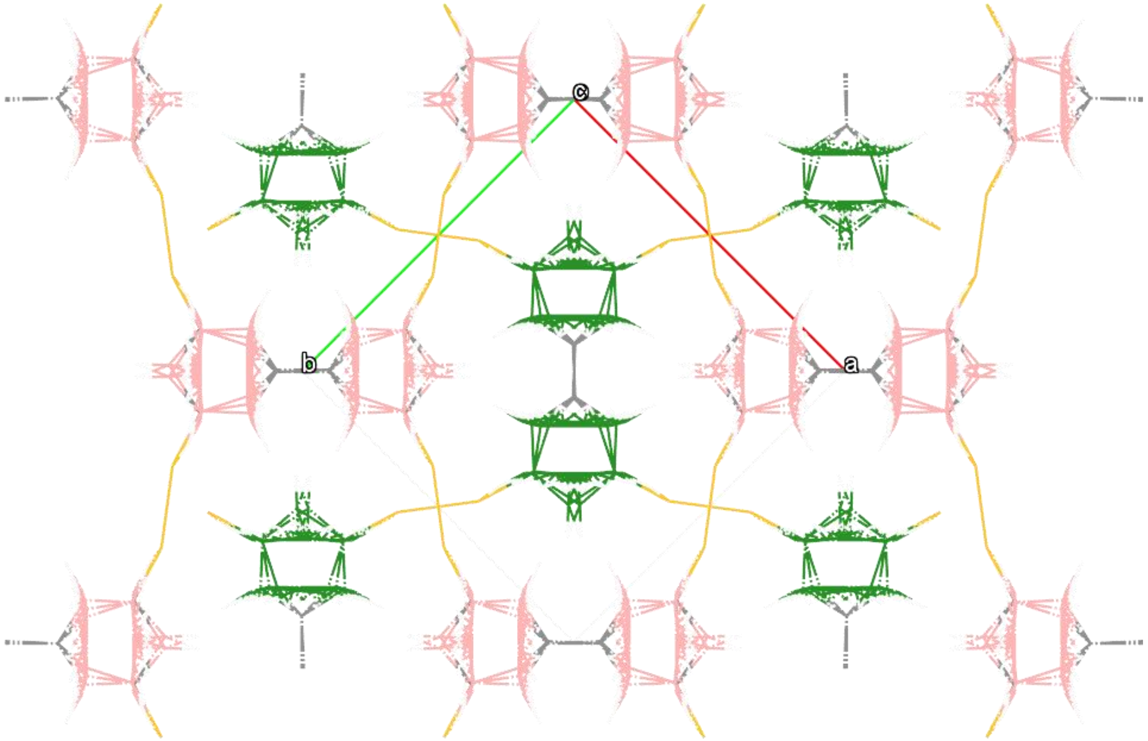

Figure S10. Stacking of 2-dimensional sheets of ***mm*-SH** molecules.

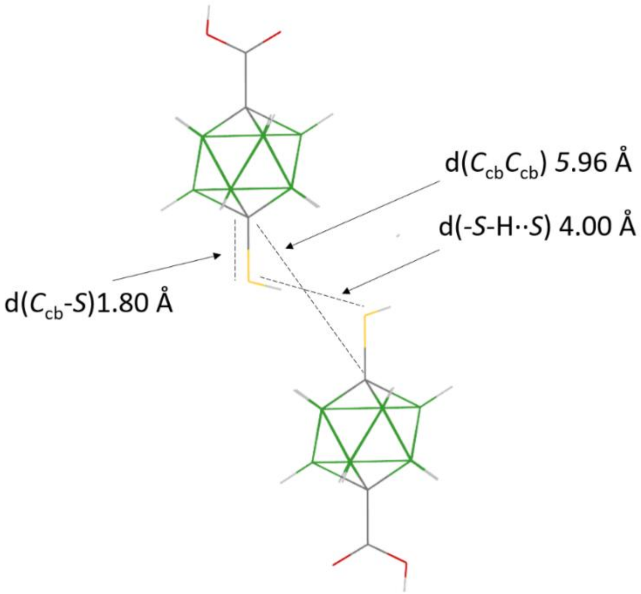

Figure S11. CCDC number 1028174.

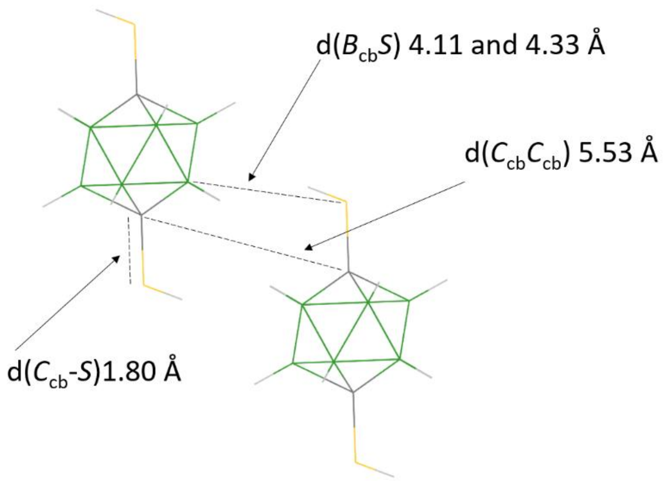

Figure S12. CCDC number 672253.

9. Dipole moments of conformers and the transition states

Table S4. Dipole moment values [D] of ***mm*-SH** rotamers and transition states.

Rotamer (Conformer) defined by the torsion angle*	Dipole moment [D]
036-minus	3.20311301
036-plus (=324-minus)	3.78319282
108-minus	1.50230411
108-plus (=252-minus)	2.98255702
180-minus	1.13184104
180-plus (=180-minus)	1.13184108

Transition State	Dipole moment [D]
000-minus (=360-minus)	3.65681115
000-plus (=360-minus)	3.65681025
072-minus	2.45917079
072-plus (=288-minus)	3.55062533
144-minus	0.64490480
144-plus (=236-minus)	2.13232338

* Torsion angle

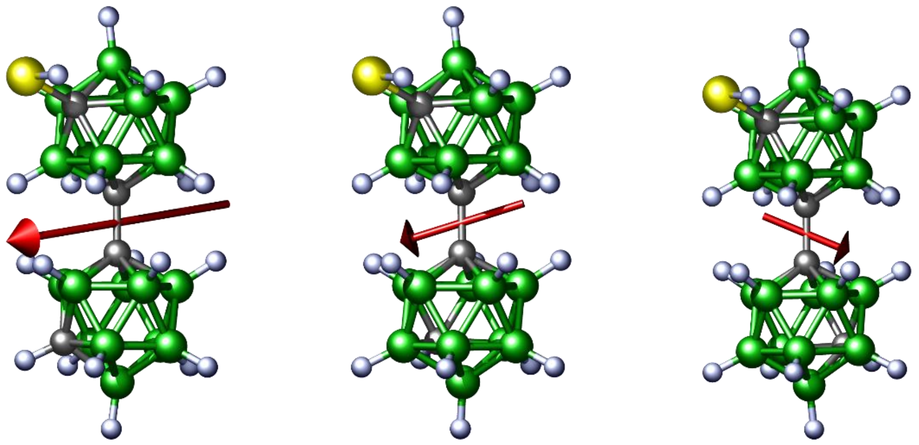

Figure S13. Graphical projection (side view) of the dipole moment vectors in three conformers of ***mm*-SH** molecule.

10. X-ray Photoelectron Spectroscopy Analysis

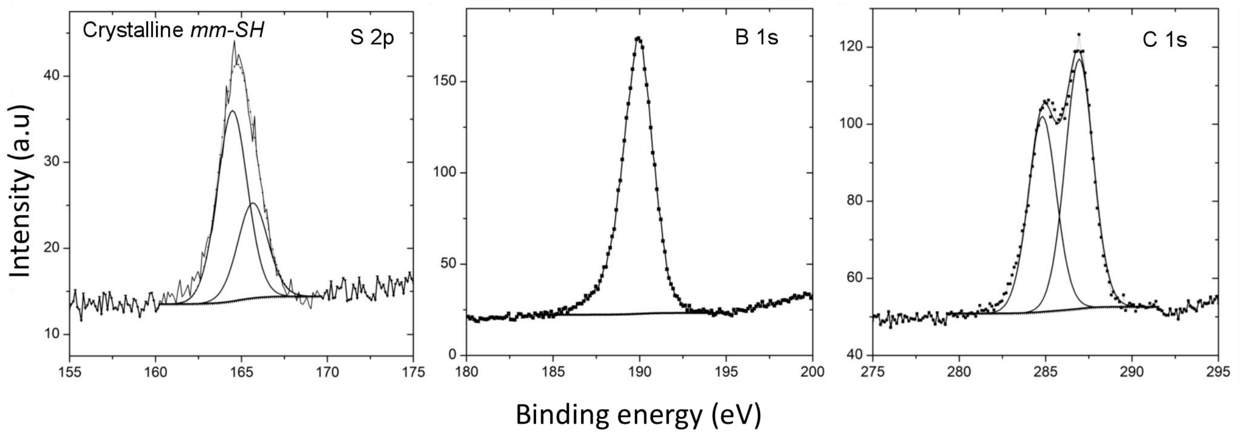

Figure S14. XP spectra of crystalline ***mm*-SH.**

11. Low-Energy Electron Diffraction Analysis

Table S5. Lattice parameters of the ***mm*-SH** SAM on Ag(111) obtained from quantitative LEED analysis. The unit cell and the corresponding LEED pattern are highlighted in blue in Figure 7b. $|\vec{a}_1|$ and $|\vec{a}_2|$ represent the lengths of the lattice vectors of the ***mm*-SH** structure with $\angle(\vec{a}_1, \vec{a}_2)$ as their enclosing angle. $\angle(\vec{a}_1, \vec{s}_1)$ denotes the angle between the first ***mm*-SH** lattice vector and the first substrate lattice vector. $\widehat{M}$ represents the resulting epitaxy matrix. The numbers in brackets indicate the uncertainties in the last digit(s).

**Structure**	$|\vec{a}_1|$ **[Å]**	$|\vec{a}_2|$ **[Å]**	$\angle(\vec{a}_1, \vec{a}_2)$ **[°]**	$\angle(\vec{a}_1, \vec{s}_1)$ **[°]**	$\widehat{M}$
***mm*-SH** SAM on Ag(111)	6.83(7)	11.47(11)	109.46(8)	10.66(7)	$\begin{pmatrix} 0.508(9) & 2.571(9) \\ 3.967(16) & -0.009(13) \end{pmatrix}$
